# MdSnRK1.1 interacts with MdGLK1 to regulate abscisic acid-mediated chlorophyll accumulation in apple

**DOI:** 10.1093/hr/uhad288

**Published:** 2023-12-29

**Authors:** Yu-Ying Yang, Xiu-Hong An, Lin Rui, Guo-Dong Liu, Yi Tian, Chun-Xiang You, Xiao-Fei Wang

**Affiliations:** State Key Laboratory of Crop Biology, Apple Technology Innovation Center of Shandong Province, Shandong Collaborative Innovation Center of Fruit & Vegetable Quality and Efficient Production, College of Horticulture Science and Engineering, Shandong Agricultural University, Tai-An 271018, Shandong, China; Key Laboratory of Chinese Herbal Medicine Biology and Cultivation, Ministry of Agriculture and Rural Affairs, Institute of Chinese Herbal Medicine, Hubei Academy of Agricultral Science, Enshi 445000, China; National Engineering Research Center for Agriculture in Northern Mountainous Areas, Agricultural Technology Innovation Center in Mountainous Areas of Hebei Province, Hebei Agricultural University, Baoding 071000, Hebei, China; State Key Laboratory of Crop Biology, Apple Technology Innovation Center of Shandong Province, Shandong Collaborative Innovation Center of Fruit & Vegetable Quality and Efficient Production, College of Horticulture Science and Engineering, Shandong Agricultural University, Tai-An 271018, Shandong, China; State Key Laboratory of Crop Biology, Apple Technology Innovation Center of Shandong Province, Shandong Collaborative Innovation Center of Fruit & Vegetable Quality and Efficient Production, College of Horticulture Science and Engineering, Shandong Agricultural University, Tai-An 271018, Shandong, China; National Engineering Research Center for Agriculture in Northern Mountainous Areas, Agricultural Technology Innovation Center in Mountainous Areas of Hebei Province, Hebei Agricultural University, Baoding 071000, Hebei, China; State Key Laboratory of Crop Biology, Apple Technology Innovation Center of Shandong Province, Shandong Collaborative Innovation Center of Fruit & Vegetable Quality and Efficient Production, College of Horticulture Science and Engineering, Shandong Agricultural University, Tai-An 271018, Shandong, China; State Key Laboratory of Crop Biology, Apple Technology Innovation Center of Shandong Province, Shandong Collaborative Innovation Center of Fruit & Vegetable Quality and Efficient Production, College of Horticulture Science and Engineering, Shandong Agricultural University, Tai-An 271018, Shandong, China

## Abstract

Abscisic acid (ABA), as a plant hormone, plays a positive role in leaf chlorosis; however, the underlying molecular mechanism is less known. Our findings provide ABA treatment reduced the chlorophyll accumulation in apple, and *Malus × domestica* Sucrose Non-fermenting 1-Related Protein Kinase 1.1 (*MdSnRK1.1)* participates in the process. MdSnRK1.1 interacts with MdGLK1, a GOLDEN2-like transcription factor that orchestrates development of the chloroplast. Furthermore, MdSnRK1.1 affects MdGLK1 protein stability through phosphorylation. We found that Ser468 of MdGLK1 is target site of MdSnRK1.1 phosphorylation. *MdSnRK1.1*-mediated phosphorylation was critical for MdGLK1 binding to the target gene *MdHEMA1* promoters. Collectively, our results demonstrate that ABA activates MdSnRK1.1 to degrade MdGLK1 and inhibit the accumulation of chlorophyll. These findings extend our understanding on how MdSnRK1.1 balances normal growth and hormone response.

## Introduction

Due to the existence of abiotic stress, the survival of plants has been posed to a serious threaten [1]. As a stress signal, abscisic acid (ABA) plays an important role in the defense against abiotic stress [2]. Further study revealed that the ABA signaling regulatory network is very complicated, and the main ABA signaling pathway is comprised of a series of modification proteins, such as pyrabactin resistance/pyr1-like/regulatory components of ABA receptor (PYR/PYL/RCARs), type 2C protein phosphatases (PP2Cs), and SNF1-related protein kinase 2 s (SnRK2s). ABA binds to PYR and promotes interactions between the ABA-bound receptors and PP2Cs [3, 4]. With the decrease of PP2C activity, the inhibition of SnRK2 by PP2Cs also decreases, thereby reducing or eliminating the effect of PP2Cs on SnRK2. Subsequently, the freed SnRK2 phosphorylates downstream factors to enhance the expression of stress- or ABA-induced genes [5–7]. As the orthologous gene of *SnRK2*, *SnRK1* performs similar functions in the regulation of the ABA signaling pathway. PP2Cs interact with the SnRK1 catalytic subunit when ABA content is deficient. These PP2Cs dephosphorylate and inactivate SnRK1, thus participating in the ABA hormone signal pathway [8, 9].

SnRK1 is an evolutionarily conserved heterotrimeric protein kinase complex including a catalytic α-subunit (SnRK1.1/1.2/1.3 in *Arabidopsis*) and regulatory β- and γ-subunits [10–12]. SnRK1 functions as a Ser/Thr protein kinase that phosphorylates downstream proteins through a highly conserved T-loop residue (T175 in SnRK1.1) [13]. Phosphorylation of SnRK1 is crucial to activate downstream transcription factors. As an energy effect factor, SnRK1 is often associated with coordinating various stressors [14]. The kinase activity of SnRK1 is activated at low energy levels such as darkness, hunger, low nutrition, and osmotic stress [15, 16]. SnRK1 triggers massive transcriptional and metabolic processes by phosphorylating metabolic signaling molecules or enzymes to respond to low energy levels [17]. Previous reports have shown that SnRK1 is associated with the ABA pathway through stress and metabolic signals [14]. Evidence shows that SnRK1 has directly participated in ABA signaling in *Arabidopsis* [18]. In tomato seeds, ABA differentially regulates the expression of SNF1-related kinase complex genes [19]. The repressed *SnRK1* gene leads to pea embryo maturation defects, which is a phenotype similar to abscisic acid-insensitive [20]. The synthesized peptides of conserved motifs of ABA-responsive basic leucine zippers (bZIPs) [21], the recombinant abscisic acid-insensitive 5 (ABI5) and bZIP12 (also known as EEL or DPBF4) proteins can be phosphorylated by SnRK1 in *Arabidopsis* [22]. bZIP63 is a downstream substrate of the SnRK1 catalytic subunit in *Arabidopsis* [23]. Previous studies showed that *MdSnRK1.1* transgenic apple materials are extremely sensitive to ABA treatment [24]. Once ABA activates MdSnRK1.1, MdSnRK1.1 phosphorylates and affects the protein stability of MdCAIP1 [24].

In the growth and development process of *Arabidopsis*, ABA treatment induces leaf yellowing, reduces chlorophyll levels, and impairs chloroplast development [25, 26]. In *Arabidopsis*, the elevation of ABA levels leads to the low expression of chlorophyll biosynthesis genes, such as Glu tRNA reductase (*HEMA1*) and protochlorophyllide oxidoreductase (*POR*) [27]. Moreover, the activity of constitutive photomorphogenic 1 (COP1) decreases with the suppression of chlorophyll content after long periods of ABA treatment [28]. Specific protein interaction between COP1 and GLK1 has been found to play pivotal roles during the ABA treatment process [28].

**Figure 1:**
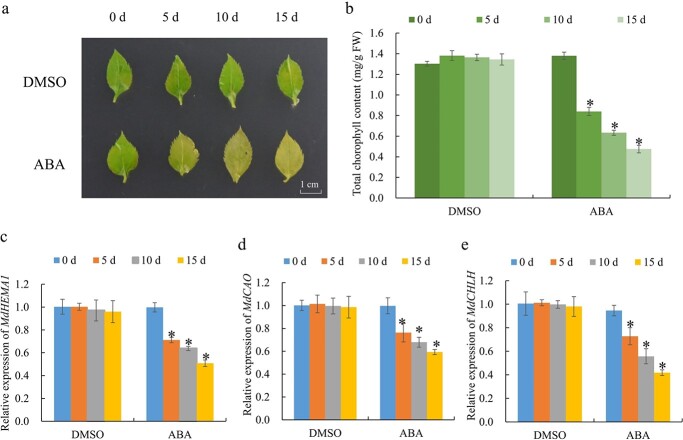
Inhibition of chlorophyll accumulation under ABA treatment. **a** Phenotypes of GL-3 apple seedling leaves in the presence or absence of 150 μM ABA for 15 days. Scale bars, 1 cm. **b** Chlorophyll contents of GL-3 apple seedling leaves treated with or without ABA for 15 days. **c**–**e** Relative expression of *MdHEMA1*, *MdCAO*, and *MdCHLH* in GL-3 apple seedling leaves treated with or without ABA for 15 days. The expression levels before the treatment (DMSO, 0 days) might be set to 1. Values are mean ± SD of three biological replicate experiments and asterisks denote a significant difference compared to the control: ^*^P < 0.05.

GLK is a member of the v-myb avian myeloblastosis viral oncogene homolog (MYB) transcription factor family, which protein contains a C terminal GCT-box and a highly conserved DNA binding domain (DBD) [29]. GLK transcription factors are involved in the regulation of chlorophyll accumulation and the synthesis of nuclear chloroplast-localized proteins in plants [29–31]. In *Arabidopsis*, the size of the chloroplast and the numbers of thylakoid lamellae in *glk1glk2* double mutants are both smaller than normal growth strain [14]. In tomato, the high expression of *SlGLK2* affects the chloroplast development and photosynthetic efficiency [32, 33].

In this study we demonstrated that ABA treatment inhibited the accumulation of chlorophyll in apple leaves. We propose a potential model to describe the role of MdSnRK1.1. in the modulation of ABA response. The protein kinase MdSnRK1.1 participated in the apple ABA signaling pathway by interacting with MdGLK1 which is a chloroplast development-related protein. MdSnRK1.1 phosphorylated MdGLK1 and reduced its protein stability. Accordingly, this study uncovered the mechanism of how MdSnRK1.1-MdGLK1 regulates ABA-modulated chlorophyll accumulation in apple.

## Results

### ABA inhibits the accumulation of chlorophyll in apple leaves

ABA is confirmed to be involved in various abiotic stress processes in plants [2]. Long-term application of ABA leads to chlorosis of plant leaves [25, 26]. To observe the phenotype of apple leaves under ABA treatment, apple leaves were treated with 0, 25, 50, 100, 150, 200, and 300 μM ABA for 0 to 30 days, and dimethyl sulfoxide (DMSO) was used as a control. We found that severe damage occurred to apple leaves after ABA treatment at the concentration of 200 and 300 μM. The most significant phenomenon of leaf chlorosis occurred when ABA concentration was 150 μM, and the total chlorophyll content was also the lowest (Fig. S1, see online supplementary material). The chlorophyll content and leaf color did not change after 15 days of ABA treatment compared to 30 days of ABA treatment. Therefore, 150 μM ABA concentration treatment for 15 days was used for subsequent apple leaf treatment. Chlorosis of apple leaves began to appear as ABA treatment time was extended (Fig. 1a). The chlorophyll content of leaves also gradually decreased as ABA treatment time was extended compared to the DMSO treatment group (Fig. 1b). The transcript level of the chlorophyll biosynthesis genes *MdHEMA1*, *MdCAO*, and *MdCHLH* also decreased gradually (Fig. 1c–e). The 15-day ABA-treated yellow plants turned green when transferred to DMSO plates (Fig. S2a, see online supplementary material). Correspondingly, the chlorophyll content recovered to the level of continuous DMSO treatment for 30 days (Fig. S2b, see online supplementary material). Therefore, the yellowing phenotype of apple leaves induced by ABA was caused by insufficient chlorophyll accumulation, which affected chloroplast development.

### *MdSnRK1.1* transcription is induced by ABA

**Figure 2:**
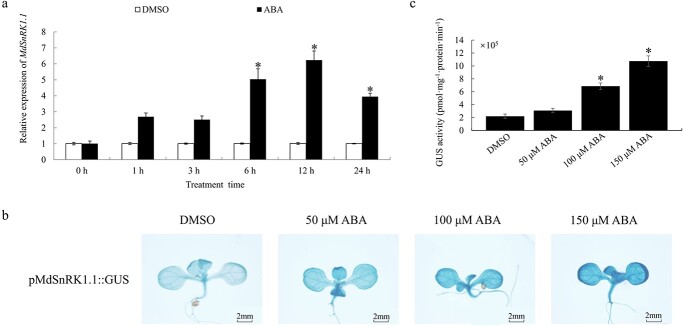
The ABA response of *MdSnRK1.1*. **a***MdSnRK1.1* expression in GL-3 apple seedlings treated with ABA. The expression levels before the treatment (DMSO, 0 hours) might be set to 1. Values are mean ± SD of three biological replicate experiments and asterisks denote significant differences compared to the control: ^*^P < 0.05. **b**–**c** The pMdSnRK1.1::GUS transgenic *Arabidopsis* treated with 0, 50, 100, or 150 μM ABA. Scale bars, 2 mm.

SnRK1 participates in ABA signaling network in *Arabidopsis* [9]. To clarify whether *MdSnRK1.1* directly involves in ABA signaling pathway, the transcription level of *MdSnRK1.1* was examined in ABA-treated apple plants. The expression of *MdSnRK1.1* was detected when the GL-3 samples were treated with ABA at 0, 1, 3, 6, 12, and 24 h. The transcription level of *MdSnRK1.1* was induced under ABA treatment, and induction was most obvious at 12 h (Fig. 2a). pMdSnRK1.1::GUS transgenic *Arabidopsis* plants were obtained to further detect the impact of ABA on the transcription level of *MdSnRK1.1* (Fig. S3a, see online supplementary material). The GUS staining of ABA-treated pMdSnRK1.1::GUS seedlings deepened as the ABA concentration was increased (Fig. 2b). The GUS activity of the ABA-treated pMdSnRK1.1::GUS seedlings was higher than DMSO-treated seedlings (Fig. 2c). These results indicated that ABA induced the transcription level of *MdSnRK1.1*.

### *MdSnRK1.1* inhibits ABA-modulated chlorophyll accumulation

**Figure 3:**
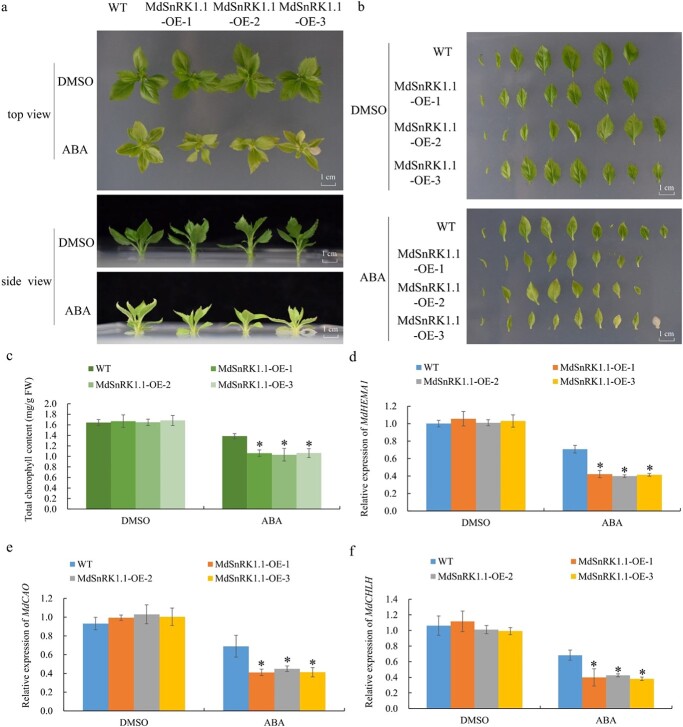
MdSnRK1.1 plays an essential role in ABA-induced leaf yellowing. **a**, **b** Phenotypes of the WT and MdSnRK1.1-OE apple seedlings under the ABA treatment. Scale bars, 1 cm. **c** Chlorophyll contents of the WT and MdSnRK1.1-OE apple seedlings under the ABA treatment. **d**–**f** Relative expression of *MdHEMA1*, *MdCAO*, and *MdCHLH* in WT and MdSnRK1.1-OE apple seedling plants after treated with ABA. The expression levels in WT treated with DMSO might be set to 1. Values are mean ± SD of three biological replicate experiments and asterisks denote significant differences compared to the control: ^*^P < 0.05; ^**^P < 0.01.

Three HIS-MdSnRK1.1 transgenic apple seedling lines were obtained for further study, and were identified at the gene expression level, the transcript level, and the protein level to ensure the accuracy of the experimental results (Fig. S3b–d, see online supplementary material). MdSnRK1.1-OE and GL-3 seedlings were treated with or without ABA to elucidate how MdSnRK1.1 responds to chlorophyll accumulation under ABA treatment. These results showed that MdSnRK1.1-OE plants were more sensitive to ABA than the wild type, with decreased chlorophyll content (Fig. 3a–c). Transcript levels of *MdHEMA1*, *MdCAO*, and *MdCHLH* decreased in MdSnRK1.1-OE plants after ABA treatment (Fig. 3d–f), suggesting that MdSnRK1.1 was involved in the reduction of chlorophyll content caused by ABA treatment. To further support this result, we performed the experiment that *MdSnRK1.1* transgenic *Arabidopsis* treated with ABA. As a result, compared with Col, the MdSnRK1.1-OE *Arabidopsis* seedlings gradually showed reduced biomass and chlorophyll content along with the decreased transcript levels of chloroplast developmental-related genes under ABA treatment (Fig. S4a–f, see online supplementary material). Therefore, these data indicated that MdSnRK1.1 regulated the ABA-modulated suppression of chlorophyll accumulation. In *Arabidopsis*, SnRK1 responds to ABA signaling pathway [9]. The expression of the homologs genes in ABA signaling (including *MdABI1* and *MdABI2* belong to PP2Cs) [34] in apple were recused by ABA and the *MdSnRK1.1* overexpression (Fig. S5a and b, see online supplementary material), and the transcripts of the overlapping genes of *MdSnRK1.1* [including MDP0000173500 (*MdSnRK1.2*) and MDP0000320932 (*MdSnRK1.3*)] [35] in apple were induced by ABA and the *MdSnRK1.1* overexpression (Fig. S5c and d, see online supplementary material).

**Figure 4:**
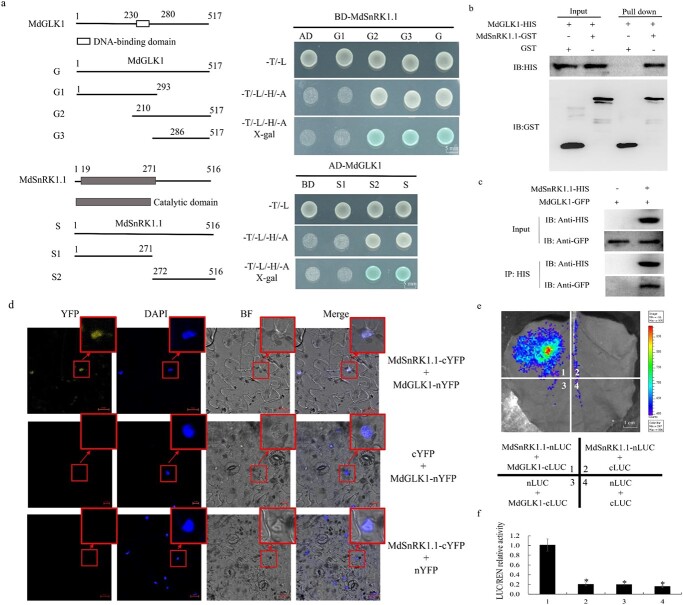
MdSnRK1.1 interacts with the MdGLK1 protein. **a** Yeast two-hybrid assay to examine the interaction between MdSnRK1.1 and MdGLK1. Scale bars, 5 mm. **b***In vitro* pull-down assay indicating the interaction between MdSnRK1.1 and MdGLK1. **c** Co-IP assay indicating the interaction between MdSnRK1.1 and MdGLK1. **d** BiFC assay in *Nicotiana benthamiana* leaves showing the interaction between MdSnRK1.1 and MdGLK1. Scale bars, 50 μm. **e**–**f** The dual luciferase assay was performed in *N. benthamiana* leaves to verify the interaction between MdSnRK1.1-nLUC and MdGLK1-cLUC. Scale bars, 1 cm.

**Figure 5:**
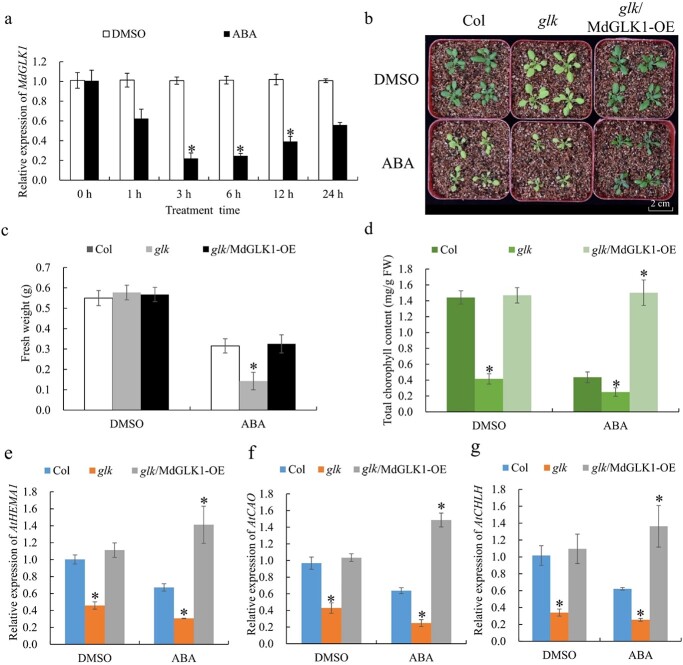
MdGLK1 is involved in ABA-induced leaf yellowing. **a** The expression of *MdGLK1* by qRT-PCR in GL-3 seedlings treated with ABA. The expression levels before the treatment (DMSO, 0 hours) might be set to 1. **b** Phenotypes of the *glk* mutant and the *glk*/MdGLK1-OE *Arabidopsis* complementation line. Scale bars, 2 cm. **c** Fresh weight of the *glk* mutant and the *glk*/MdGLK1-OE *Arabidopsis* complementation line. **d** Chlorophyll contents of the *glk* mutant and the *glk*/MdGLK1-OE *Arabidopsis* complementation line. **e**–**g** Relative expression of *AtHEMA1*, *AtCAO*, and *AtCHLH* in the *glk* mutant and *glk*/MdGLK1-OE *Arabidopsis* complementation line. The expression levels in Col treated with DMSO might be set to 1. Values are mean ± SD of three biological replicate experiments and asterisks denote significant differences compared to the control: ^*^P < 0.05; ^**^P < 0.01.

### MdSnRK1.1 interacts with the MdGLK1 protein

In order to explore the molecular mechanism of MdSnRK1.1 participating in ABA-modulated chlorophyll accumulation, a Y2H screen was performed using MdSnRK1.1 as bait to identify any potentially interacting proteins. As a result, the chloroplast development related protein MdGLK1 was screened and used as a potential interaction protein for subsequent research. *MdSnRK1.1* contained a typical catalytic subunit, and *MdGLK1* contained a DNA binding domain. In order to determine the accurate interaction segment between *MdSnRK1.1* and *MdGLK1*, we divided its sequence into several segments based on their structural domain. The *MdSnRK1.1* gene was divided into two segments to construct the pGBD-MdSnRK1.1-S (amino acids 1–516), pGBD-MdSnRK1.1-S1 (amino acids 1–271), and pGBD-MdSnRK1.1-S2 (amino acids 272–516) yeast vectors. The *MdGLK1* gene was divided into three segments to construct pGAD-MdGLK1-G (amino acids 1–517), pGAD-MdGLK1-G1 (amino acids 1–293), pGAD-MdGLK1-G2 (amino acids 210–517), and pGAD-MdGLK1-G3 (amino acids 286–517) yeast vectors. The Y2H assay showed that the S2 segment of *MdSnRK1.1* physically interacted with the G3 segment of *MdGLK1* (Fig. 4a). Pull-down assays were carried out by using the MdSnRK1.1-GST, MdGLK1-HIS, and GST fusion proteins. These results showed that MdGLK1-HIS protein was enriched by MdSnRK1.1-GST. In contrast, the MdGLK1-HIS protein was not enriched by the GST control (Fig. 4b), indicating that MdSnRK1.1 physically interacted with MdGLK1. The Co-IP assay was performed to confirm MdSnRK1.1 interact with MdGLK1 *in vivo* (Fig. 4c). Then, a BiFC assay was conducted to further confirm this result. The full-length cDNA fragment of *MdSnRK1.1* was cloned to the C-terminal half of the YFP to construct MdSnRK1.1-cYFP, and the *MdGLK1* gene sequence was cloned to the N-terminal half of YFP to construct MdGLK1-nYFP. Consistently, fluorescence signals were only observed in the nucleus of *Nicotiana benthamiana* leaves cells when MdSnRK1.1-cYFP and MdGLK1-nYFP were co-expressed (Fig. 4d). Lastly, dual luciferase assays were carried out in *N. benthamiana* leaves to confirm the interaction. The full-length sequences of *MdSnRK1.1* and *MdGLK1* were inserted into the pGreenII 0800-LUC vector to obtain the reporter constructs. Four combination vectors, including MdSnRK1.1-nLUC+MdGLK1-cLUC, MdSnRK1.1-nLUC+cLUC, nLUC+MdGLK1-cLUC, and nLUC+cLUC were injected into *N. benthamiana* leaves. As a result, only the MdSnRK1.1-nLUC+MdGLK1-cLUC treatment group caused the *N. benthamiana* to fluoresce (Fig. 4e and f). Taken together, these data revealed that MdSnRK1.1 interacted with MdGLK1 *in vivo* and *in vitro*.

### The chloroplast development gene *MdGLK1* responds to ABA treatment

To test whether MdGLK1 involves in ABA-modulated chlorophyll accumulation, the expression level of *MdGLK1* was first detected under ABA treatment. The qRT-PCR detection results showed that the expression of *MdGLK1* was obviously repressed at 3 h after ABA treatment in apple (Fig. 5a). Then, *glk* mutant and *glk*/MdGLK1-OE transgenic *Arabidopsis* were obtained to verify the possible role of MdGLK1 in regulating ABA-modulated chlorophyll accumulation. When treated with ABA, the chlorophyll content of *glk* mutant plants was lower than that of Col, and the expression of chloroplast developmental-related genes were also significantly repressed (Fig. 5b–g). However, the complementation line rescued the phenotype of leaf chlorosis under ABA treatment (Fig. 5b and d). Compared to the wild-type, the transcript levels of the chloroplast developmental-related genes, including *AtHEMA1*, *AtCAO*, and *AtCHLH*, decreased in the *glk* mutant but increased in *glk*/MdGLK1-OE plants. Overall, these results confirmed that MdGLK1 played a significant role in ABA-induced chlorophyll content decrease in apple.

### MdSnRK1.1 destabilizes the MdGLK1 protein

**Figure 6:**
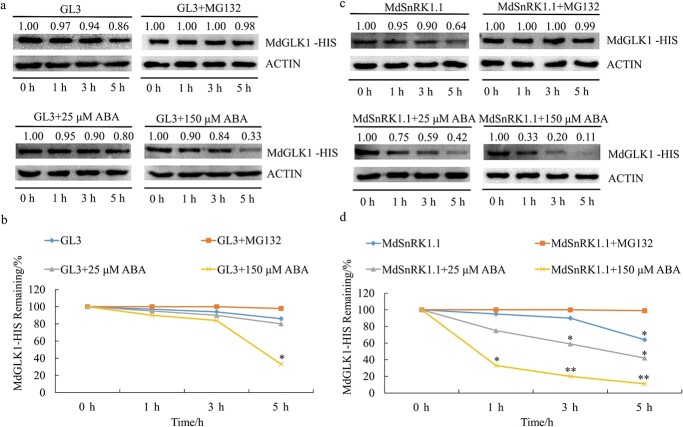
MdSnRK1.1 promotes destabilization of MdGLK1 protein. **a** The MdGLK1-HIS protein and total protein samples were extracted from GL-3 apple seedlings treated with or without ABA and incubated together at 22°C for the indicated period. **c** The MdGLK1-HIS protein and total protein samples were extracted from MdSnRK1.1-OE apple seedlings treated with or without ABA and incubated together at 22°C for the indicated period. **b**, **d** The protein levels at 0, 1, 3, and 5 h were examined in **a** and **c**.

As the key metabolic switch, the SNF1 protein kinase acts on some enzymes and transcription factors and affects protein stability by phosphorylating its substrates in yeast [36–38]. SNF1 responds to energy demand and environmental stress by inducing or repressing metabolic pathways [19]. Considering the potential kinase activity of MdSnRK1.1 and the repressed expression of *MdGLK1* under ABA conditions, we proposed that MdSnRK1.1 might affect the stability of MdGLK1 protein. An *in vitro* protein degradation assay was performed to explore the effect of MdSnRK1.1 on the protein stability of MdGLK1. The total protein extracts isolated from GL-3 with or without ABA treatment were cultured with the MdGLK1-HIS fusion protein. As a result, the MdGLK1-HIS protein level decreased significantly as ABA treatment time was extended (Fig. 6a and b). However, in the presence of MG132, the MdGLK1-HIS level was not affected by ABA treatment, which indicated the protein of MdGLK1-HIS is degraded in a 26S proteasome-dependent manner (Fig. 6a). These studies showed that ABA treatment significantly reduced the MdGLK1 protein content. To examine whether the overexpression of *MdSnRK1.1* would affect the protein level of MdGLK1 under ABA treatment, we performed the same ABA treatment on HIS-MdSnRK1.1 transgenic apple seedlings and tested the MdGLK1 protein level (Fig. 6c). The relative protein content of MdGLK1-HIS was 33% when treated with ABA for 5 h under the background of the GL-3 seedlings, while the relative protein content of MdGLK1-HIS was only 11% when treated with ABA for 5 h under the background of the HIS-MdSnRK1.1 transgenic apple seedlings (Fig. 6d). Furthermore, MdSnRK1.1-MdGLK1 transgenic apple seedlings were obtained by instantaneous transformation. The *in vivo* protein degradation assay was carried out to identify the degradation of MdGLK1 was accelerated by MdSnRK1.1 under ABA treatment (Fig. S6, see online supplementary material). All of these results indicated that MdSnRK1.1 accelerated the degradation of MdGLK1 protein.

### MdSnRK1.1 phosphorylates MdGLK1 and affects its protein stability

**Figure 7:**
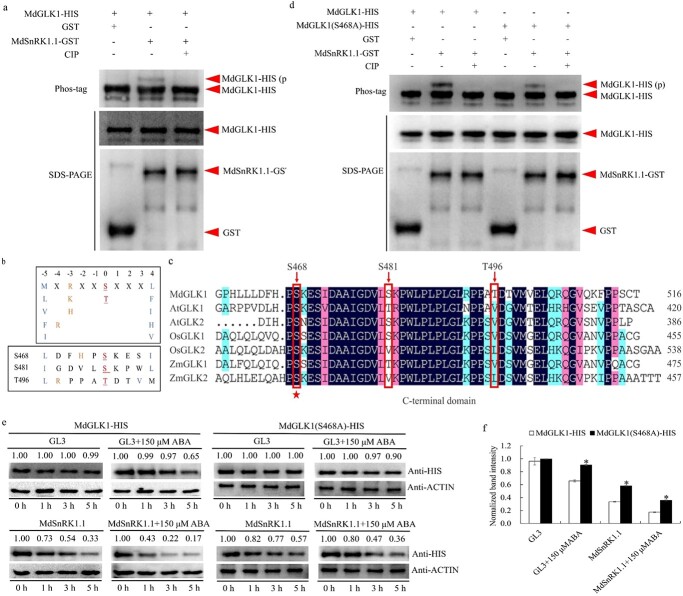
MdSnRK1.1 affects the stability of MdGLK1 protein by phosphorylating serine at 468 position of MdGLK1. **a** Modulation of MdGLK1 phosphorylation by MdSnRK1.1 *in vitro*. **b** Analysis of three potential phosphorylation sites predicted by the website. **c** Conservative analysis of potential phosphorylation sites in various species. **d** Modulation of serine 468 mutation MdGLK1 phosphorylation by MdSnRK1.1 *in vitro*. **e** The MdGLK1-HIS protein or MdGLK1(S468A)-HIS mixed with total protein samples which extracted from GL-3 apple seedlings treated with or without ABA and incubated together at 22°C for the indicated period. **f** The nomalized band intensity were examined in **e**.

SnRK1 plays its role by phosphorylating downstream substrates under normal conditions [17]. The protein phosphorylation process is often involved in the modulation of protein stability [39]. To investigate whether MdSnRK1.1 affected the protein stability of MdGLK1 through phosphorylation, we first performed *in vitro* kinase assays that combined MdSnRK1.1-GST kinase protein with the MdGLK1-HIS protein. A Phos-tag assay carried out that MdSnRK1.1 could phosphorylate MdGLK1 *in vitro* and this phosphorylation could be inhibited by phosphatase inhibitor cocktail (CIP) (Fig. 7a). To determine the potential phosphorylation site of MdGLK1, we logged on http://prosite.expasy.org/scanprosite to predict the possible phosphorylation sites of *MdGLK1* according to the recognition sequence of *SnRK1* ([MLVFI]-[XRKH]-[XRKH]-XX-[ST]-XXX-[LFIMV]). The three potential functional sites Ser468, Ser481, and Thr496 were analysed (Fig. 7b). An amino acid sequence alignment was constructed to determine the conservation of phosphorylation sites between the *MdGLK1* with other *GLK* genes from *Arabidopsis*, rice, and maize. It was found that the serine at 468 of MdGLK1 was conserved in all species (Fig. 7c). Then, a variant of MdGLK1 harboring nonphosphorylatable Ser468 to Ala468 point mutation (S468A) was generated. In the gel containing Phos-tag, the phosphorylation of MdGLK1 was significantly weakened, but could not be diminished after the serine mutation at 468 (Fig. 7d). Meanwhile, the phosphorylation of MdGLK1 was not weakened after mutations at the Ser481 and Thr496 (Fig. S7, see online supplementary material). These results suggested S468 of MdGLK1 as target site of MdSnRK1.1 phosphorylation. To investigate the influence of MdSnRK1.1 on the stability of MdGLK1 protein after phosphorylation site mutation, the protein degradation assay was performed. The serine deletion at position 468 partially blocked the degradation of MdGLK1 induced by ABA treatment, suggesting that MdSnRK1.1 promoted the degradation of MdGLK1 protein through the phosphorylation pathway (Fig. 7e–f). To verify whether other post-translational modifications of proteins, such as ubiquitination, affect the degradation of MdGLK1 protein by MdSnRK1.1, we conducted a ubiquitination test and found that MdGLK1 did not undergo ubiquitination modification (Fig. S8, see online supplementary material). It is reported that GLK1 binds to conserved promoter sequences CCAATC of *HEMA1* gene [31]. EMSA analysis was conducted to test whether MdSnRK1.1 phosphorylation affected MdGLK1 binding to target gene *MdHEMA1*. MdGLK1 bound to the probe (−454) of *MdHEMA1* promoter and the binding of MdGLK1 (S468A) to the *MdHEMA1* promoter was significantly attenuated (Fig. S9, see online supplementary material). To verify whether phosphorylated MdGLK1 promotes or inhibits the expression of *MdHEMA1* by binding to its promoter, we added a bimolecular luciferase assay. We found that the fluorescence signal of MdHEMA1pro:LUC + 35Spro:MdGLK1 (S468A) was comparable to that of MdHEMA1pro:LUC + 35Spro:62-SK, and its luciferase activity has not significantly increased, indicating that the mutation of MdGLK1 phosphorylation site inhibits its ability to activate downstream *MdHEMA1* promoter transcription (Fig. S10, see online supplementary material). In general, *MdSnRK1.1*-mediated phosphorylation was critical for MdGLK1 binding to the target gene promoters.

**Figure 8:**
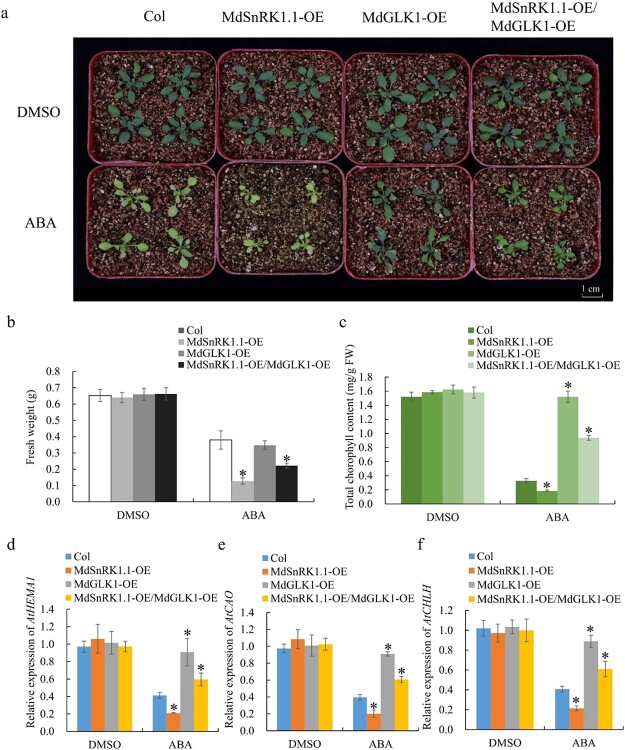
MdSnRK1.1 and MdGLK1 affect chlorophyll accumulation together. **a** Phenotypes of the MdSnRK1.1-OE, MdGLK1-OE, and MdSnRK1.1-OE/MdGLK1-OE *Arabidopsis* complementation line. Scale bars, 1 cm. (**b**) Fresh weight of McadSnRK1.1-OE, MdGLK1-OE, and the MdSnRK1.1-OE/MdGLK1-OE *Arabidopsis* complementation line. **c** Chlorophyll contents of the MdSnRK1.1-OE, MdGLK1-OE, and MdSnRK1.1-OE/MdGLK1-OE *Arabidopsis* complementation line. **d**–**f** Relative expression of *AtHEMA1*, *AtCAO*, and *AtCHLH* in the MdSnRK1.1-OE, MdGLK1-OE, and MdSnRK1.1-OE/MdGLK1-OE *Arabidopsis* complementation line. The expression levels in Col treated with DMSO might be set to 1. Values are mean ± SD of three biological replicate experiments and asterisks denote significant differences compared to the control: ^*^P < 0.05; ^**^P < 0.01.

### MdGLK1 and MdSnRK1.1 jointly regulate ABA-modulated chlorophyll accumulation

The tissue expression patterns revealed that *MdSnRK1.1* and *MdGLK1* had their highest expression levels in leaves (Fig. S11a and b, see online supplementary material). The subcellular localization analysis of *MdSnRK1.1* and *MdGLK1* showed that *MdSnRK1.1* was localized to the nucleus and cytoplasm, while *MdGLK1* was localized to the nucleus (Fig. S11c, see online supplementary material). The genetic materials of MdGLK1-OE and MdSnRK1.1-OE/MdGLK1-OE plants were obtained to further explore the genetic regulation of *MdSnRK1.1* and *MdGLK1*. These genetic materials treated with ABA showed that chlorophyll content accumulated in MdGLK1-OE under the ABA treatment, while it was reduced in MdSnRK1.1-OE plants compared to the Col (Fig. 8a). However, MdSnRK1.1-OE/MdGLK1-OE plants alleviated the MdSnRK1.1-OE yellow leaf phenotype (Fig. 8a–c), which was similar to the chlorophyll contents and the expression patterns of the chloroplast developmental genes including *AtHEMA1*, *AtCAO*, and *AtCHLH* (Fig. 8d–f). These results indicated that MdGLK1 and MdSnRK1.1 jointly regulate ABA-modulated chlorophyll accumulation.

## Discussion

The chloroplast is an important place for plants to carry out photosynthesis, amino acid metabolism, hormone biosynthesis, and other metabolic processes [40–42]. Chlorophyll is the most important pigment in plant photosynthesis. This is known as ABA is a stress hormone that induces leaf yellowing [2]. In our study, we first discussed the relationship between ABA and chlorophyll. ABA treatment affects the content of chlorophyll in apple leaves, leading to the leaves of apple seedlings turning yellow (Fig. S1, see online supplementary material). Yellowing of leaves often indicates senescence in plants. It has been previously reported that ABA treatment accelerates the senescence process of plants [43–45]. We therefore tested whether long-term ABA treatment induced leaf yellowing by promoting leaf senescence, thus clarifying the molecular mechanism of ABA-induced leaf yellowing (Fig. S2, see online supplementary material). We found that DMSO restored the leaf yellowing phenotype caused by ABA treatment. However, plant aging is irreversible [46]. We demonstrated that the treatment of ABA induced leaf yellowing by inhibiting the accumulation of chlorophyll in apple.

The ABA signal response is a complex process involving a number of genes, such as PYR, PP2Cs, and SnRK1 [3, 4]. In *Arabidopsis*, SnRK1, as an important protein kinase, participates in ABA signaling through its interaction with PP2C [9]. Overexpression of *AtSnRK1.1* in *Arabidopsis* delays the growth of seedlings and enhances the response to ABA. However, the overexpression of *AtSnRK1.1* had no effect on *Arabidopsis* seed germination [18]. It has been reported that SnRK1 increases ABA levels in pea embryos, thereby regulating ABA-mediated seed maturation [47]. However, the SnRK1 overexpression is not to alter the ABA level in *Arabidopsis* [18]. Overexpression of *MdSnRK1.1* promotes maturation of tomato fruit, and ABA plays a role in tomato ripening [48]. We found that ABA increased the *MdSnRK1.1* transcription level (Fig. 2). Previous studies have shown that MdSnRK1.1-OE apple calli leads to a highly ABA-sensitive phenotype, suggesting that MdSnRK1.1 participates in the ABA signaling pathway in apple [24]. We verified that ABA-induced leaf yellowing of MdSnRK1.1-OE seedlings was related to chlorophyll accumulation (Fig. 3). ABA-induced leaf yellowing, and overexpression of *MdSnRK1.1* aggravated this phenomenon (Fig. 3). Due to plants being unable to move, they must have various ways to cope with the constantly changing external environmental conditions. Therefore, MdSnRK1.1 plays a balanced role in ABA-mediated growth inhibition and chloroplast development-induced growth promotion. In addition, SnRK1 directly destabilizes the ETHYLENE INSENSITIVE3 (EIN3) to slow down senescence-associated leaf yellowing in *Arabidopsis* [49]. We speculated that SnRK1 jointly regulated the leaf yellowing phenotype through two hormone pathways.

In this study, we proposed a new mechanism by which MdSnRK1.1 participates in ABA-mediated leaf chlorosis through its interaction with chloroplast developmental-related protein MdGLK1 (Fig. 4). MdGLK1 was expressed in photosynthetic tissues, such as leaves, which is the same location as MdSnRK1.1 (Fig. S8, see online supplementary material). *GLK* complements the *Arabidopsis glk1glk2* double mutant, in which chloroplasts exhibit an increase in the number of thylakoid grana [50]. Here, ABA negatively regulated the expression of *MdGLK1* and accelerated degradation of the MdGLK1 protein (Figs 5a and 6a). Overexpressing *MdGLK1* attenuated the ABA-induced yellowing leaf phenotype in Col and MdSnRK1.1-OE *Arabidopsis* seedlings (Fig. 8a). These findings described that MdSnRK1.1 and MdGLK1 suppressed chlorophyll accumulation under ABA treatment.

More studies are needed to understand the relationship between MdSnRK1.1 and MdGLK1. SnRK1 is very conservative in evolution as a protein kinase. SnRK1 forms heterotrimeric complexes with the catalytic α-subunit and regulatory β and γ-subunits [10, 39, 51]. SnRK1 affects the stability of downstream kinases or transcription factors through pathways, such as phosphorylation [24, 36]. Stability of the MdGLK1 protein was affected by ABA treatment. MdGLK1 was degraded under the ABA treatment, which is consistent with the leaf yellowing caused by ABA when chlorophyll accumulation was suppressed. The protein degradation assay demonstrated that the MdGLK1 protein was less stable under the MdSnRK1.1-OE seedling condition than under the control condition after ABA treatment (Fig. 6). All of these data indicated that MdSnRK1.1 was critical for the degradation of MdGLK1, and MdSnRK1.1 inhibited the activity of MdGLK1 to induce suppression of chlorophyll accumulation under an ABA treatment.

Considering the involvement of MdGLK1 in the post-translational modification process to the protein, we speculated that it might undergo ubiquitination degradation dependent on the 26S proteasome pathway. The ubiquitination test found that MdGLK1 did not undergo ubiquitination modification (Fig. S9, see online supplementary material). In *Arabidopsis*, SnRK1 phosphorylates its specific substrates to be involved in various life processes [21]. The substrates include the key metabolic enzymes (such as sucrose phosphate synthase, nitrate reductase, and 3-hydroxy-3-meth-ylglutaryl CoA reductase), and the transcription factors (such as FUSCA3) [52–54]. AtSnRK1.1 phosphorylates and positively regulates FUSCA3 to modulate ABA responses [54, 55]. Previous studies reported that MdSnRK1.1 interacted with and phosphorylated MdCAIP1 in apple [24]. Meanwhile, we screened Ser468 of MdGLK1 as a potential target site for MdSnRK1.1 phosphorylation, and this phosphorylation site is crucial for the protein degradation of MdGLK1 by MdSnRK1.1 and the binding of MdGLK1 to the *MdHEMA1* gene promotor. Overall, our findings suggested that MdSnRK1.1 phosphorylated MdGLK1 and accelerated its degradation to involve the ABA signaling pathway, providing a regulatory mechanism for further study of ABA signaling.

## Conlusion

**Figure 9:**
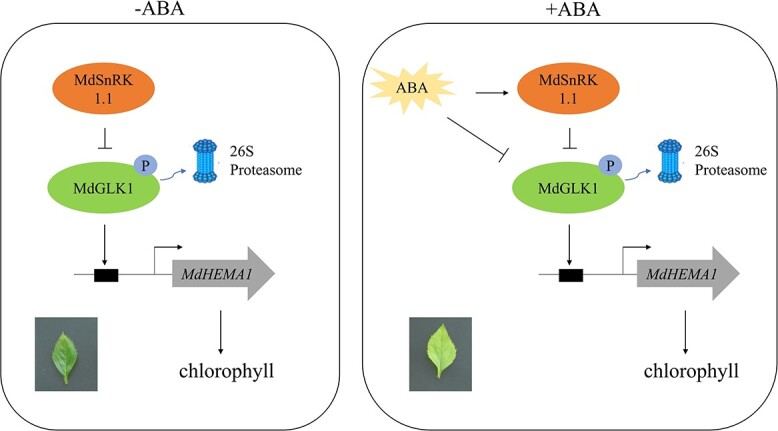
A model of *MdSnRK1.1*-mediated chlorophyll accumulation in response to ABA in apple.

Taken together, our data provide a mechanism of the ABA-modulated chlorophyll accumulation in apple (Fig. 9). MdSnRK1.1 represses the ABA-regulated chlorophyll accumulation by direct protein–protein interaction with MdGLK1 and inhibits the transcriptional activation of downstream target gene *MdHEMA1* by MdGLK1. Under ABA deficiency conditions, the transcription level of *MdSnRK1.1* is reduced, and the phosphorylation of MdSnRK1.1 on MdGLK1 is weakened, which contributes to chlorophyll accumulation. In the condition of ABA, MdSnRK1.1 contributes to the degradation of the MdGLK1 protein via the 26S proteasome pathway, inhibiting the transcriptional activation of the *MdHEMA1* by MdGLK1, thereby inhibiting the accumulation of chlorophyll.

## Materials and methods

### Plant materials and ABA treatment

The selection of the wild-type tissue cultures is *Malus* × *domestica* cv ‘GL-3’ apples and *Arabidopsis thaliana* ‘Columbia’ ecotype (Col). The *glk* mutant was obtained from the European *Arabidopsis* Stock Centre (NASC ID N9807). The ‘GL-3’ and MdSnRK1.1-OE seedlings were cultured on Murashige and Skoog (MS) medium supplemented with 0.5 mg/L gibberellic acid (GA), 0.2 mg/L α-naphthaleneacetic acid (NAA), and 1 mg/L 6-benzyladenine (6-BA), and Col was cultured on MS medium [56]. For ABA treatments, the 30-day-old apple seedlings with the same grown period were treated on medium added with ABA for 15 days at 22°C under a 16/8 h light/dark period. The 15-day-old *Arabidopsis* seedlings were growth on medium added with 150 μM ABA for 15 days at 22°C under a 16/8 h light/dark period for the ABA treatment.

### Acquisition of transgenic plants

The open reading frames (ORFs) of the *MdSnRK1.1* and *MdGLK1* genes were cloned into the pRI101 vector. The overexpressing plasmids were constructed by using *Agrobacterium* (LBA4404)-mediated transformation that transformed the MdSnRK1.1-pRI recombinant plasmid into ‘GL-3’ cultures [57]. The transgenic lines were selected on MS medium that contained 250 mg/L timentin, 0.5 mg/L NAA, 2 mg/L thidiazuron (TDZ), and 25 mg/L kanamycin for apple seedlings for 30 days under a dark period. For *Arabidopsis* transformation, the vectors were genetically transformed into Col with *Agrobacterium tumefaciens* strain GV3101 using the floral dip transformation method [58], and the third generation of transformed *Arabidopsis* plants was analysed. For *N. benthamiana* agrobacterium-mediated infiltration, the vectors were genetically transformed into GV3101 until the culture grew to saturation. The precipitate was mixed with the solution containing 100 mM acetosyringone, 25 mM MgCl_2_, and 25 mM MES and kept at 25°C for 4 h, and then injected into *N. benthamiana* leaves using a needleless syringe.

### Quantitative real-time polymerase chain reaction (qRT-PCR)

The RNAplant plus Reagent (Tiangen, Beijing, China) system was used to extract the total RNA that was isolated from apple and *Arabidopsis* plants. The PrimeScript First Strand cDNA Synthesis Kit (Takara, Beijing, China) was used to compose the first-strand cDNA. The iCycler iQ5 Detection System (Bio-Rad, Hercules, CA, USA) was used to conduct the qRT-PCR reactions using the SYBR Green method. The 18S in apple was used as the loading control and each operation was performed as technical replicates three times [59]. All primers used in this study are presented in Table S1 (see online supplementary material).

### β-Glucuronidase (GUS) staining

A 2 kb promoter sequence of *MdSnRK1.1* was cloned into the pCAMBIA1300 vector to obtain pMdSnRK1.1::GUS construct, and the floral dip transformation method was used to obtain pMdSnRK1.1::GUS transgenic *Arabidopsis* [58]. For histochemical staining, pMdSnRK1.1::GUS seedlings were submerged in GUS staining buffer (0.05 mM ferricyanide, 1 mM 5-bromo-4-chloro-3-indolyl-β-glucuronide, 10 mM EDTA,100 mM sodium phosphate, 0.1% Triton X-100, 20% methanol, pH 6.8) at 37°C for 12 h, followed by de-staining with absolute ethanol for 12 h to histochemical staining [60].

### Measurement of chlorophyll content

A total of 1 g of *Arabidopsis* or apple seedling leaves was dipped into 95% ethanol in the dark for 1 day at 24°C to extract chlorophyll. A spectrophotometer was used to colorimetrically calculate the absorbance at 649 nm and 665 nm when the leaves turned white. Chlorophyll content (mg/g FW) = (20.8 × A645 + 8.04 × A663) × V/M. (A: absorbance; V: volume of alcohol; M: weight of leaves).

### Analysis of subcellular localization

The *MdSnRK1.1* and *MdGLK1* coding sequences were contracted into the green fluorescent protein (GFP) vector. The 35S::GFP-MdSnRK1.1, 35S::GFP-MdGLK1, and the 35S:GFP were transferred into the GV3101 strain. The expression vectors were infiltrated into *N. benthamiana* cells using the *Agrobacterium*-mediated method and grown for 4 days. The expression of the GFP fluorescent protein in the cells was captured by a high-resolution confocal microscope (LSCM; Carl Zeiss, Oberkochen, Germany).

### Protein degradation assay

The method of protein degradation assay was based on the paper published by An *et al.* [57]. The *in vitro* protein was induced by 0.3 mM isopropyl β-D-thiogalactoside (IPTG) from *E. coil* cells for 5 h at 37°C. The *in vivo* proteins were extracted from the transgenic apple seedlings in degradation buffer containing 2 mM phenylmethylsulfonylfluoride (PMSF), 4 mM DTT, 8 mM NaCl, 12 mM MgCl_2_, 15 mM ATP, and 25 mM Tris–HCl. The mixed solutions were detected at 0, 1, 3, and 5 h, and used HIS, GFP, or ACTIN as detection antibody.

### Yeast two-hybrid (Y2H) assay

To screen the proteins that interact with MdSnRK1.1, the ORFs of *MdSnRK1.1* was transferred into the pGBD vector as bait (pGBD-MdSnRK1.1). The Oebiotech Company (Shanghai, China) has constructed the apple cDNA library that was extracted from apple skin. Y2H screening was screened on the selective medium SD/−Leu/−Trp/-His/−Ade/5-bromo-4-chloro-3-indolyl-α-D-galactopyranoside (SD/−T/−L/-H/−A/X-gal), and the positive clones were determined by sequencing. The chloroplast-related regulatory factor MdGLK1 from the positive clones as the potential component for our following study. The specific sequences of *MdSnRK1.1* were fused into the pGBD vector (pGBD-MdSnRK1.1-S, pGBD-MdSnRK1.1-S1, and pGBD-MdSnRK1.1-S2), and the specific sequences of *MdGLK1* were fused into the pGAD vector (pGAD-MdGLK1-G, pGAD-MdGLK1-G1, pGAD-MdGLK1-G2, and pGAD-MdGLK1-G3). These conducts were confused into the Y2H Gold yeast strain and cultured on SD/−T-L and SD/−T-L-H-A selective medium at 28°C for 2 days. Through the lack of -T/−L/-H/−A medium and the addition of X-gal medium to confirm the interaction.

### Dual luciferase assay

The ORFs of *MdSnRK1.1* and *MdGLK1* were transferred into the pGreenII 0800-nLUC vector and the pGreenII 0800-cLUC vector, respectively, to contract the MdSnRK1.1-nLUC and MdGLK1-cLUC. The mixture of MdSnRK1.1-nLUC, MdGLK1-cLUC, nLUC, and cLUC were inserted into *N. benthamiana* cells. After 3 days, a live-cell image was used to measure luminescence. Detection of the LUC/REN activity was with the luciferase reporter gene detection kit (Sigma, Shanghai, China).

### Pull-down assay

The full-lengths of *MdSnRK1.1* and *MdGLK1* cDNA were transferred into the pGEX-4 T and pET-32a vector, respectively. These plasmids were transformed into *Escherichia coli* BL21 (DE3). Magnetic bead method was used to mix the induced fusion proteins, and the precipitate was detected with HIS or GST antibodies (Abmart, Shanghai, China).

### Bimolecular fluorescence complementation (BiFC) assay

The sequence of *MdSnRK1.1* was inserted to the C-terminal half of the yellow fluorescent protein (cYFP) to contract the MdSnRK1.1-cYFP and the sequence of *MdGLK1* was inserted to the N-terminal half of YFP to contract the MdGLK1-nYFP. These recombinant plasmids were inserted into the *N. benthamiana* epidermal cells, then grown for 3 days. The signal of YFP fluorescent protein in cells was captured by LSCM.

### Coimmunoprecipitation (co-IP) assay

MdGLK1-GFP and MdGLK1-GFP/MdSnRK1.1-HIS transgenic materials were applied for the co-immunoprecipitation (Co-IP) assays. An IP Kit (Thermo Fisher Scientific, Waltham, MA, USA) was used to immunoprecipitate MdSnRK1.1-HIS protein that was treated by 100 μM MG132 for 24 h, and the precipitate was detected with HIS or GFP antibodies (Abmart, Shanghai, China).

### *In vitro* phosphorylation assay

The MdGLK1-HIS protein and the MdSnRK1.1-GST kinase protein were cultured in a 20 ml reaction buffer containing 1 mM DTT, 8 mM MgCl_2_, 25 mM ATP, and 50 mM Tris–HCl. The solution was cultured at 30°C for 2 h and terminated by adding protein loading buffer. Subsequently, the samples were separated on 10% Phos-tag SDS-PAGE gels supplied with 100 μM Phos-tag (Nard, Shanghai, China) and 50 mM MnCl_2_, and detected with HIS or GST antibodies (Abmart, Shanghai, China).

### Electrophoretic mobility shift assay (EMSA)

The glutathione sepharose beads (Thermo Fisher, Scientific, Waltham, MA, USA) were used to purify the MdGLK1-HIS protein. The EMSA probe biotin labeling kit (Beyotime, Shanghai, China) was used to label the probe of the *MdHEMA1* promoter. The competitors are the same sequences without labels. The purified protein and labeled probe were mixed at 25°C for 40 min and separated on nondenaturing polyacrylamide gels.

### Data presentation and statistical analysis

All assays were repeated at least three times. DPS software was used to analyse all data statistical analysis (Digital Processing Systems, Hicksville, NY, USA). We marked *P*-values <0.05 as significant and *P*-values <0.01 as very significant.

## Acknowledgements

We thank Professor Zhi-Hong Zhang of Shenyang Agricultural University for supplying the GL-3 apple. This work was supported by the National Natural Science Foundation of China (32172538, 32272683), the National Key Research and Development Program (2018YFD1000200), China Agriculture Research System of MOF and MARA (CARS-27), Taishan Scholar Foundation of Shandong Province (LJNY202026), the Natural Science Foundation of Hebei Province (C2021204134).

## Author Contributions

C.-X.Y., X.-F.W., and Y.-Y.Y. conceived the research; X.-F.W. and C.-X.Y. performed the experiments; Y.-Y.Y., X.-H.A., L.R., G.-D.L. and Y.T. analysed most data of the experiments; X.-F.W. and Y.-Y.Y. wrote the article.

## Data availability statement

All relevant data can be found within the paper and its supporting materials.

## Conflict of interests

The authors have no conflicts of interest.

## Supplementary information

Supplementary data is available at *Horticulture Research* online.

## Supplementary Material

Web_Material_uhad288
