# Supplementary material for: MdSnRK1.1 interacts with MdGLK1 to regulate abscisic acid-mediated chlorophyll accumulation in apple
Source: Hortic Res. 2023 Dec 29;11(2):uhad288. doi: 10.1093/hr/uhad288 (PMC10873579; doi:10.1093/hr/uhad288)
Supplement: Web_Material_uhad288 [file web_material_uhad288.zip › Supplementary File for Review.pdf]

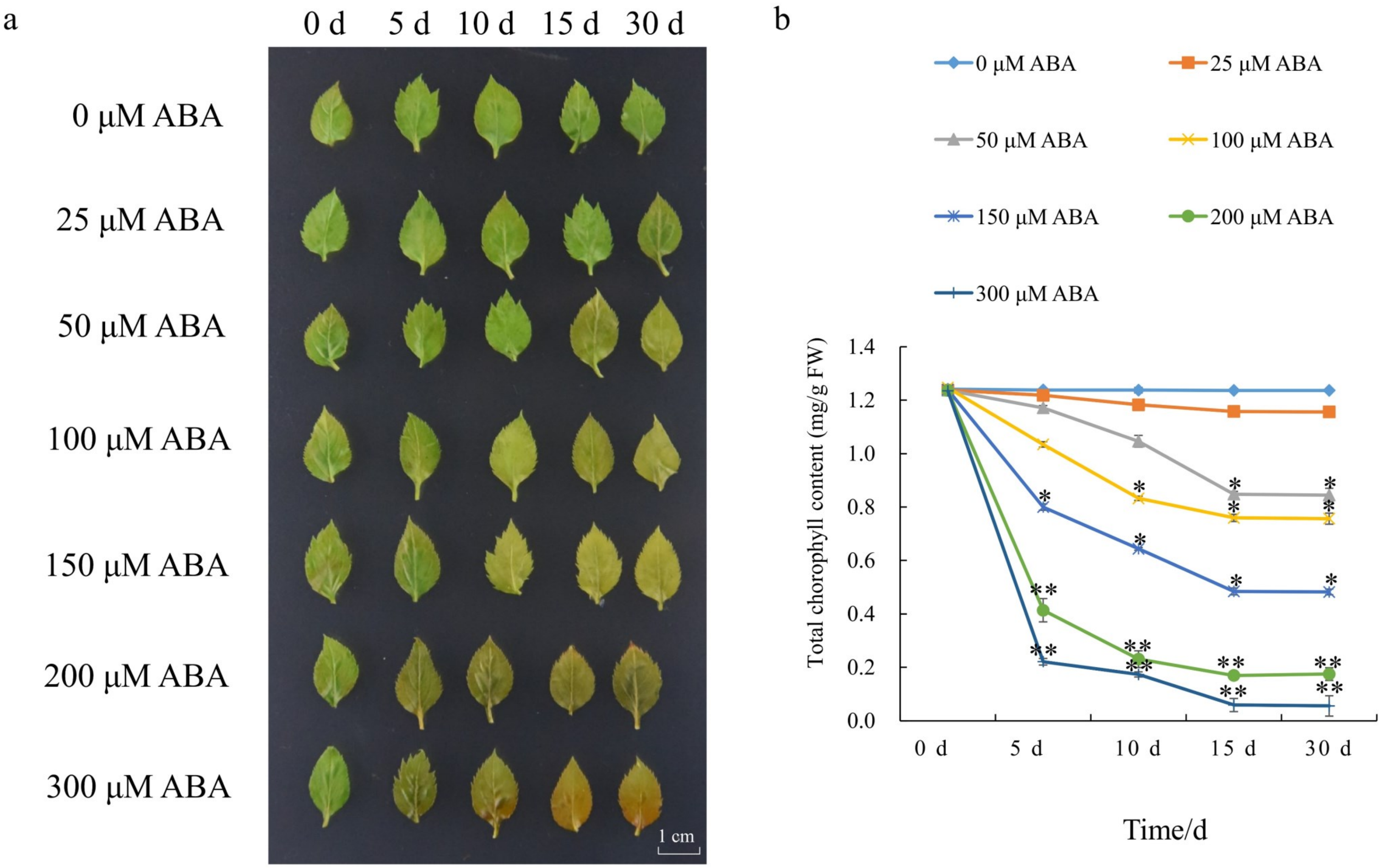

Supplemental Figure 1. Apple leaves treated with 0, 25, 50, 100, 150, 200 and 300 µM ABA for a long time. (a) Phenotypes of GL-3 apple seedling leaves in 0, 25, 50, 100, 150, 200 and 300 µM ABA for 30 days. Scale bars, 1 cm. (b) Chlorophyll contents of GL-3 apple seedling leaves treated with ABA for 30 days. Values are mean ± SD of three biological replicate experiments and asterisks denote a significant difference compared to the control: *$P < 0.05$.

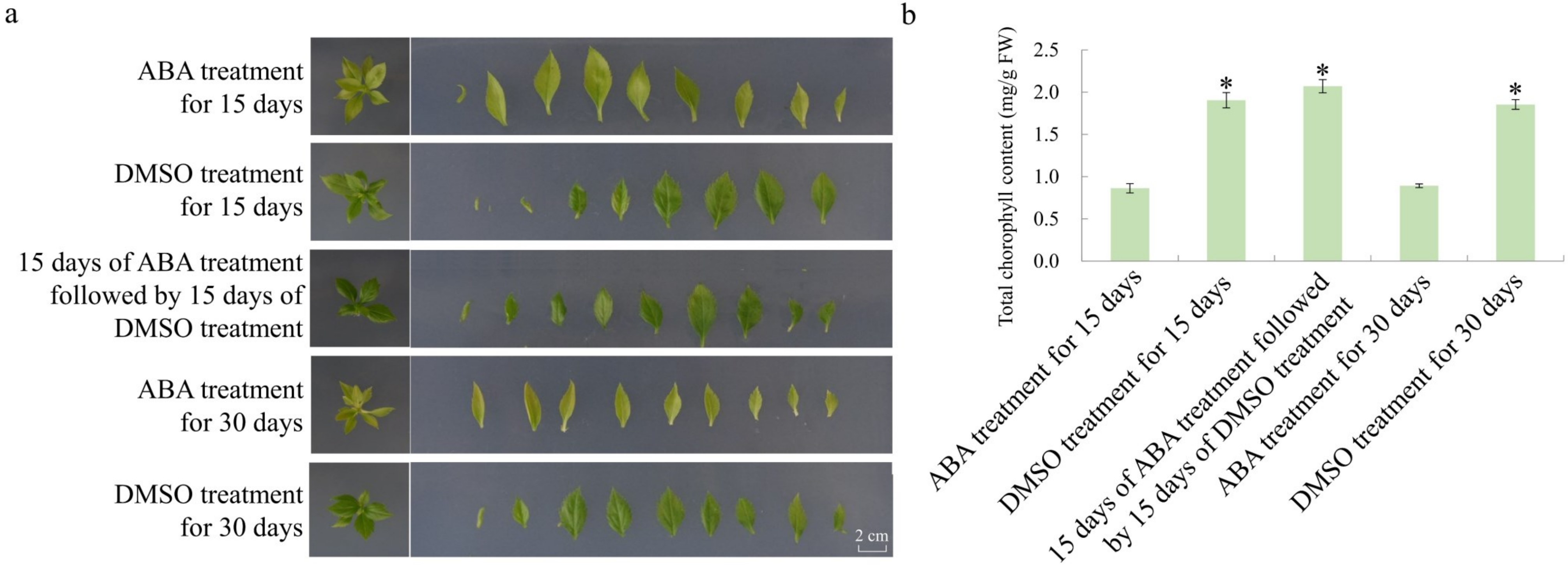

Supplemental Figure 2. Recovery of the yellow leaf phenotype of apple after transferring from ABA-containing plates to DMSO plates. (a) Phenotypes of GL-3 apple seedling with ABA or DMSO for a certain number of days. Scale bars, 2 cm. (b) Chlorophyll contents of GL-3 apple seedling leaves treated with ABA or DMSO. Values are mean ± SD of three biological replicate experiments and asterisks denote a significant difference compared to the control: *$P < 0.05$.

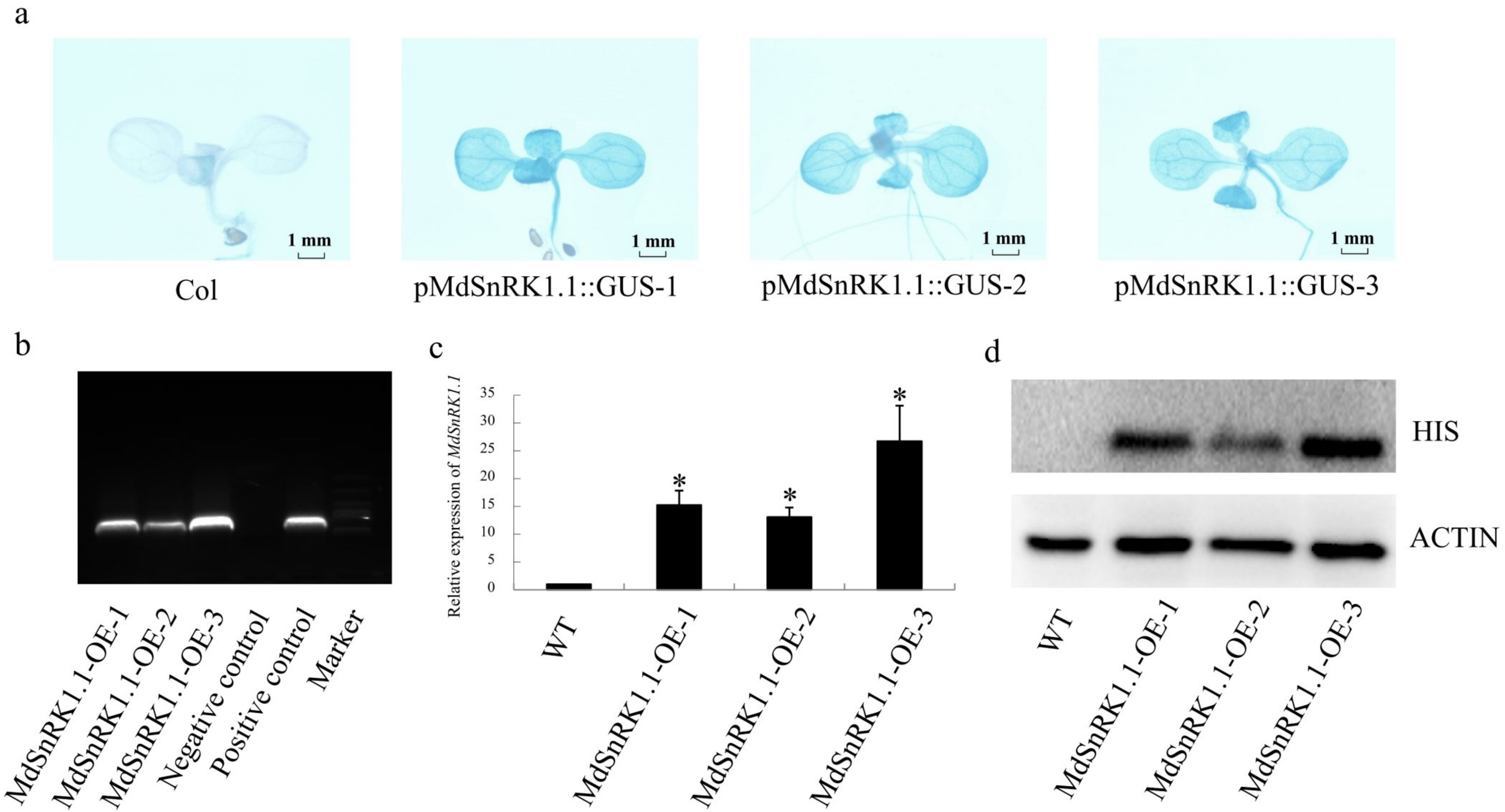

Supplemental Figure 3. Identification of MdSnRK1.1 transgenic seedlings. (a) The GUS screening of Col and three pMdSnRK1.1::GUS transgenic *Arabidopsis* plants. Scale bars, 1 mm. (b-d) The identification of three HIS-MdSnRK1.1 transgenic apple seedling lines at the DNA, RNA, and protein levels, respectively.

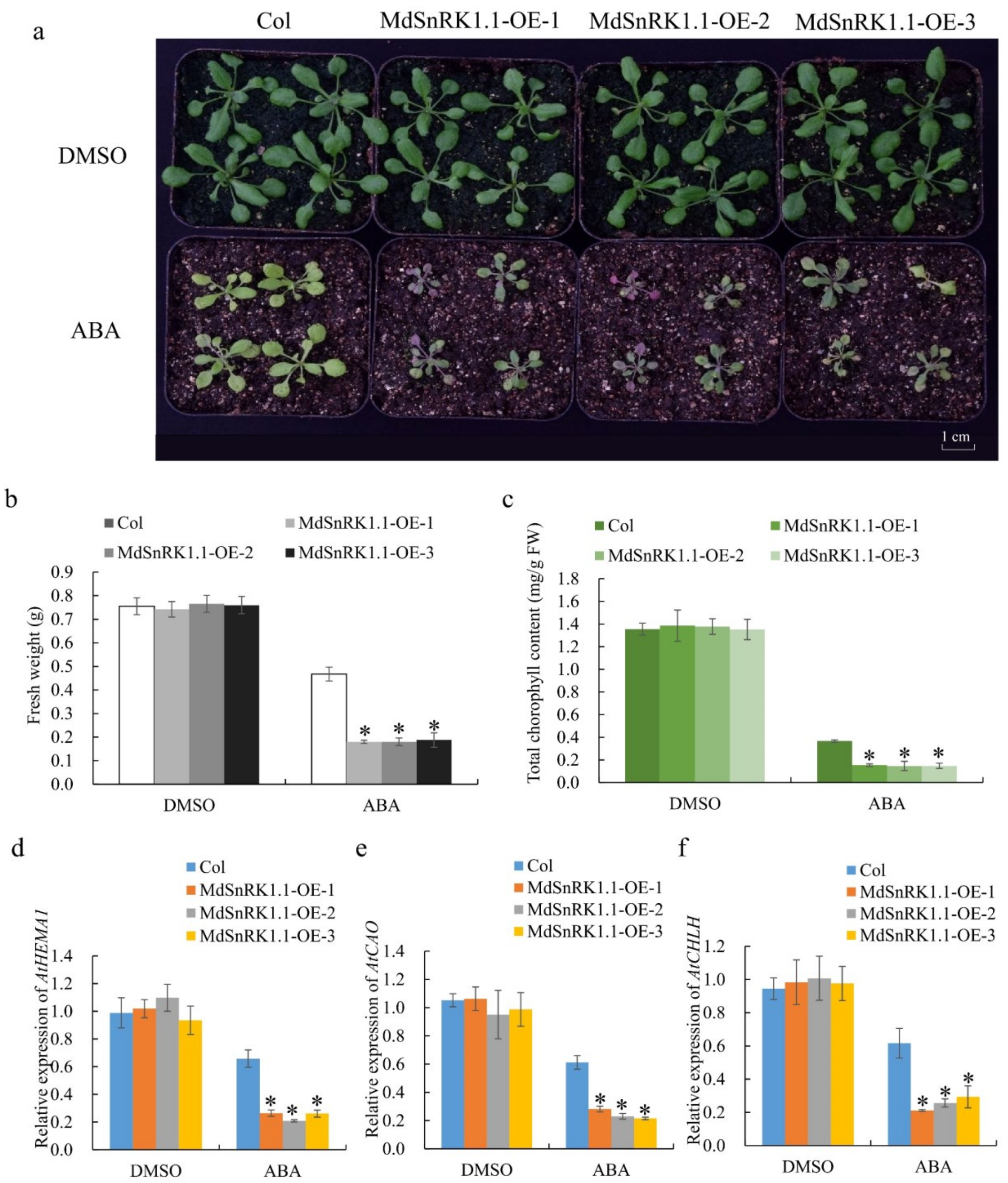

Supplemental Figure 4. Overexpression of *MdSnRK1.1* treated with ABA in *Arabidopsis*. (a, b) Phenotypes of the Col and MdSnRK1.1-OE *Arabidopsis* seedlings under the ABA treatment. Scale bars, 1 cm. (c) Chlorophyll contents of the Col and MdSnRK1.1-OE *Arabidopsis* seedlings under the ABA treatment. (d-f) Relative expression of *AtHEMA1*, *AtCAO*, and *AtCHLH* in Col and MdSnRK1.1-OE *Arabidopsis* seedlings plants after treatment with ABA. The expression level in Col treated with DMSO might be set to 1. Values are mean ± SD of three biological replicate experiments and asterisks denote significant differences compared to the control: *P < 0.05; **P < 0.01.

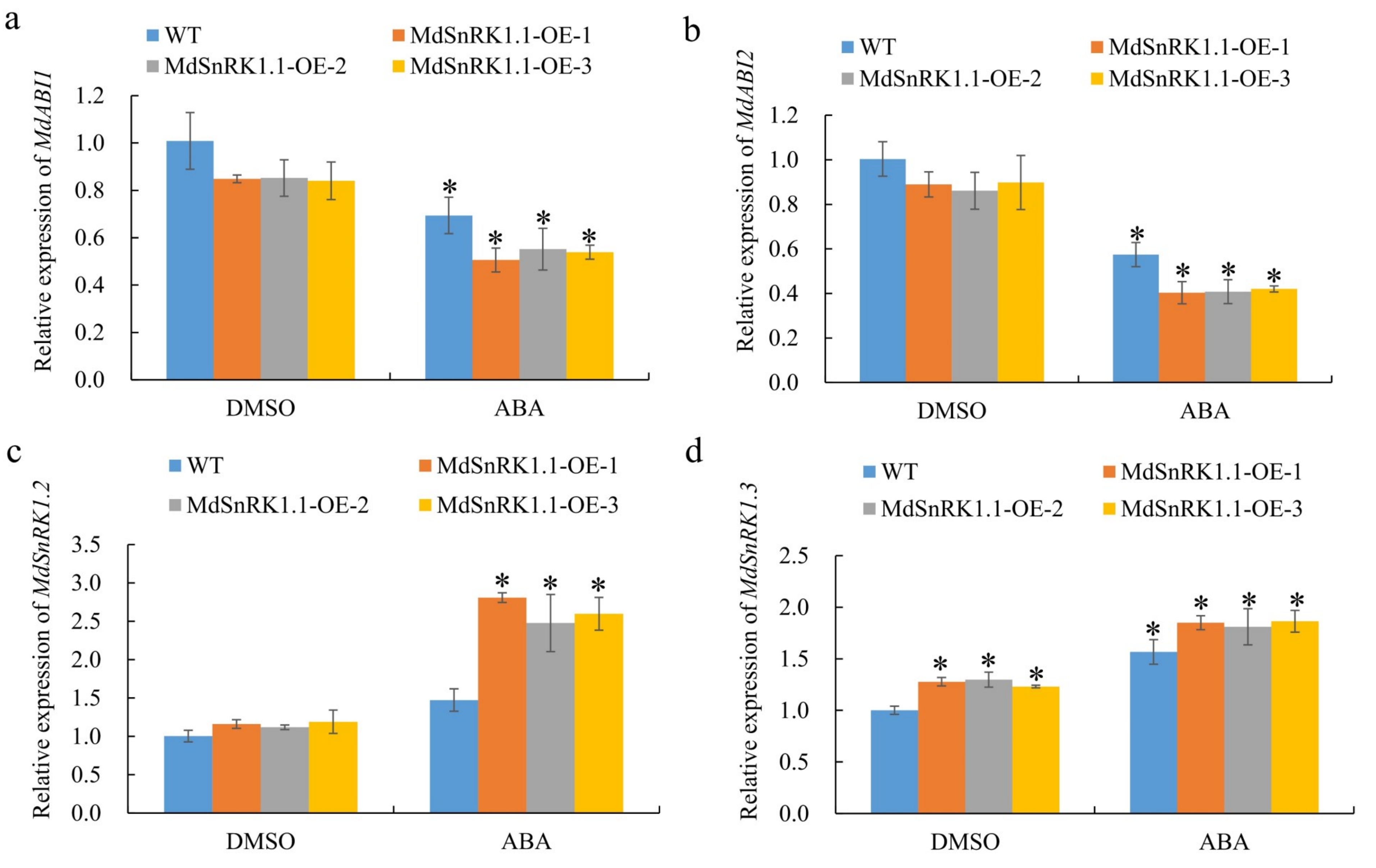

Supplemental Figure 5. The expression of *MdABI1*, *MdABI2*, *MdSnRK1.2* and *MdSnRK1.3* treated with ABA and the *MdSnRK1.1* overexpression. (a, b) The expression of *MdABI1* and *MdABI2* treated with ABA and the *MdSnRK1.1* overexpression. (c-e) The expression of *MdSnRK1.2* and *MdSnRK1.3* treated with ABA and the *MdSnRK1.1* overexpression. The expression level in WT treated with DMSO might be set to 1. Values are mean ± SD of three biological replicate experiments and asterisks denote significant differences compared to the control: *$P < 0.05$; **$P < 0.01$.

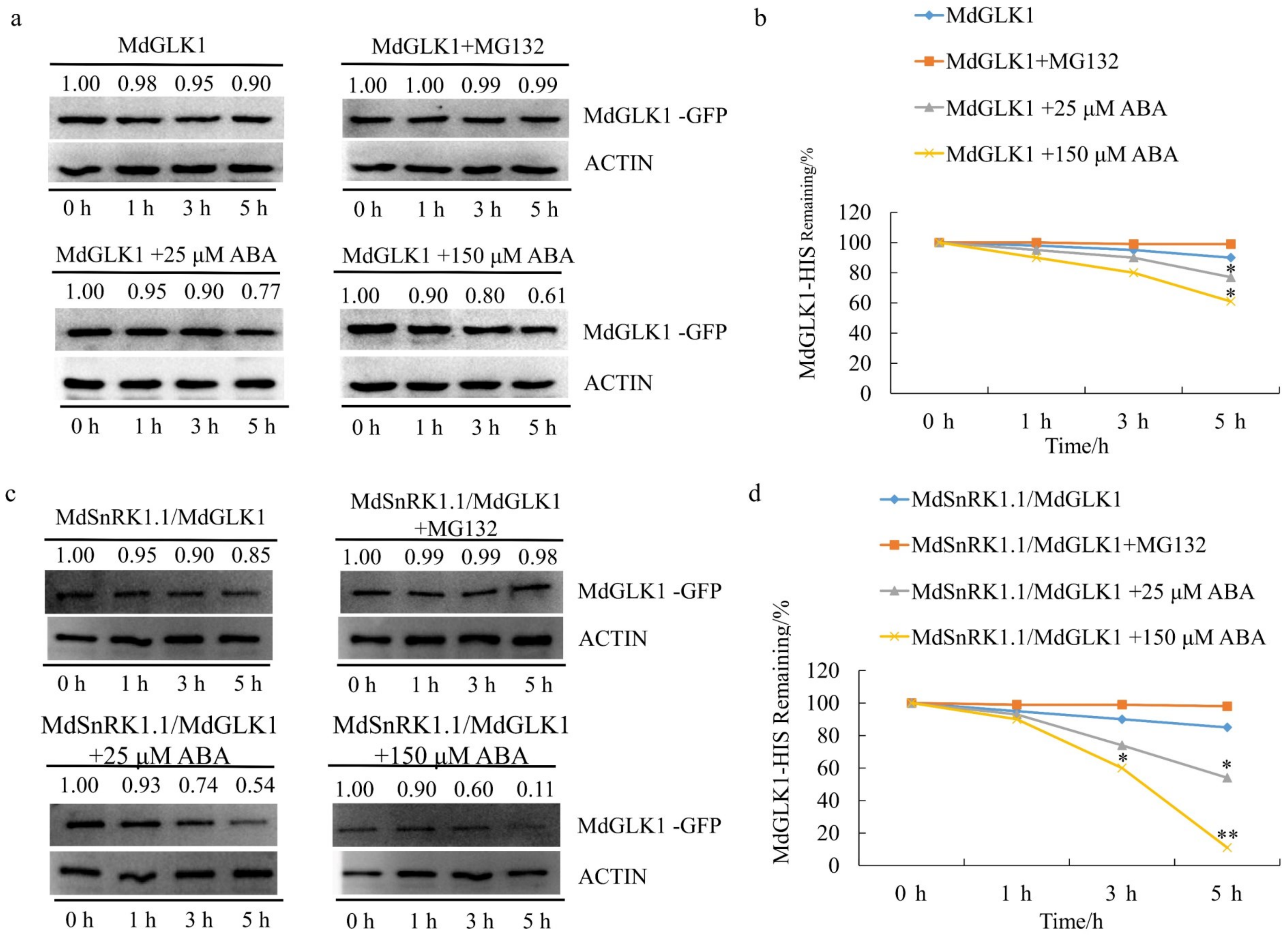

Supplemental Figure 6. MdSnRK1.1 promotes destabilization of MdGLK1 *in vivo*. (a) The MdSnRK1.1-MdGLK1 transgenic apple seedlings treated MG132 or 25 or 150 μM ABA and incubated together at 22°C for the indicated period. (b) The protein levels at 0, 1, 3, and 5 h were examine in (a). (c) The MdSnRK1.1-MdGLK1 transgenic apple seedlings treated with MG132 or 25 or 150 μM ABA and incubated together at 22°C for the indicated period. (b) The protein levels at 0, 1, 3, and 5 h were examine in (c).

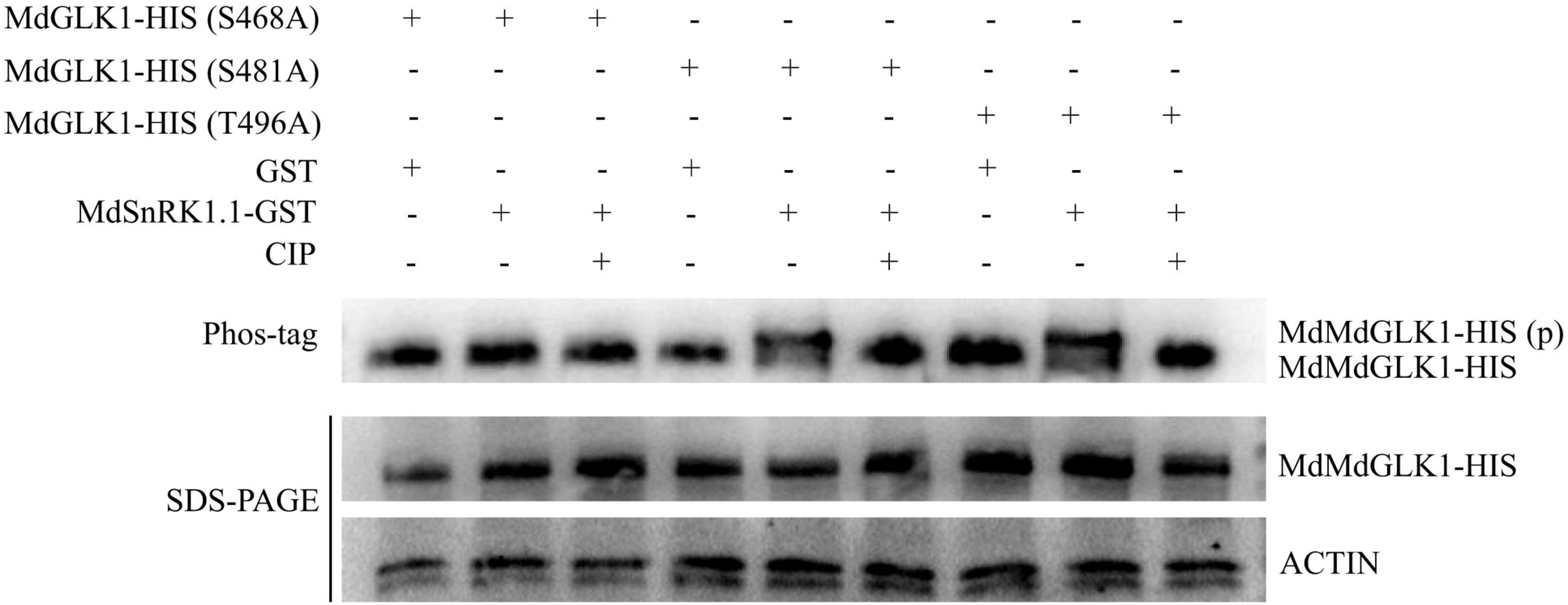

Supplemental Figure 7. Modulation of Ser468, Ser481 and Thr496 mutation MdGLK1 phosphorylation by MdSnRK1.1 *in vitro*.

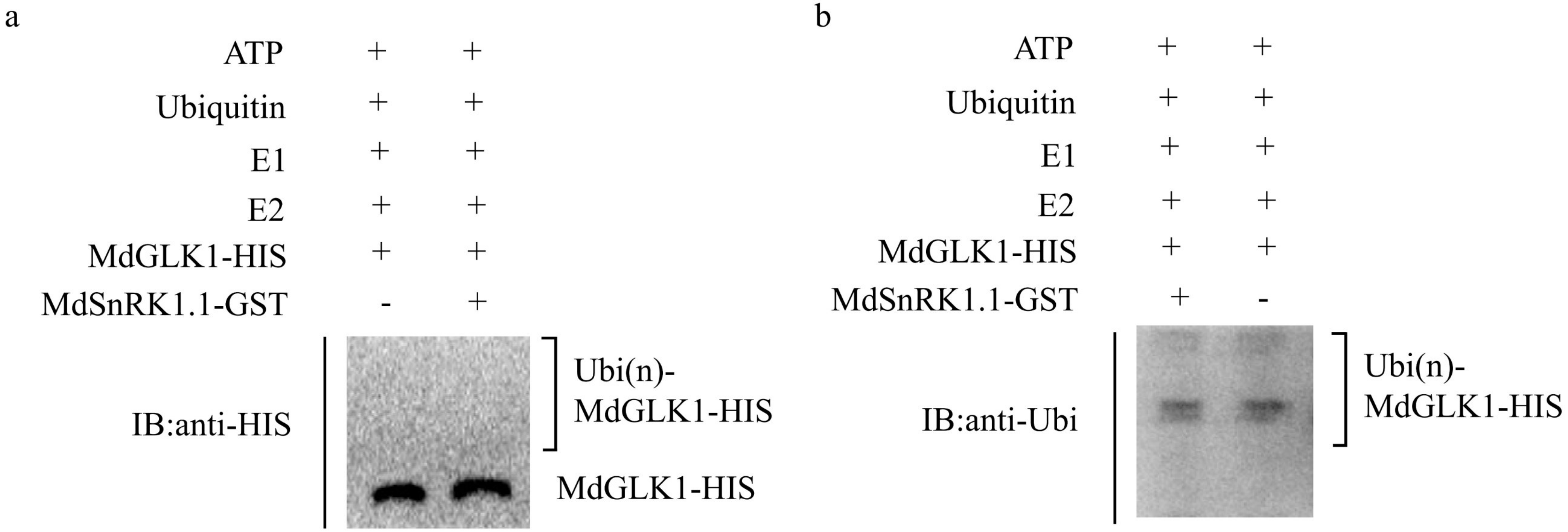

Supplemental Figure 8. Ubiquitination detection of MdGLK1 protein by MdSnRK1.1. (a) Ubiquitination of MdGLK1-HIS protein detected by anti-HIS antibody. (b) Ubiquitination of MdGLK1-HIS protein detected by anti-Ubi antibody.

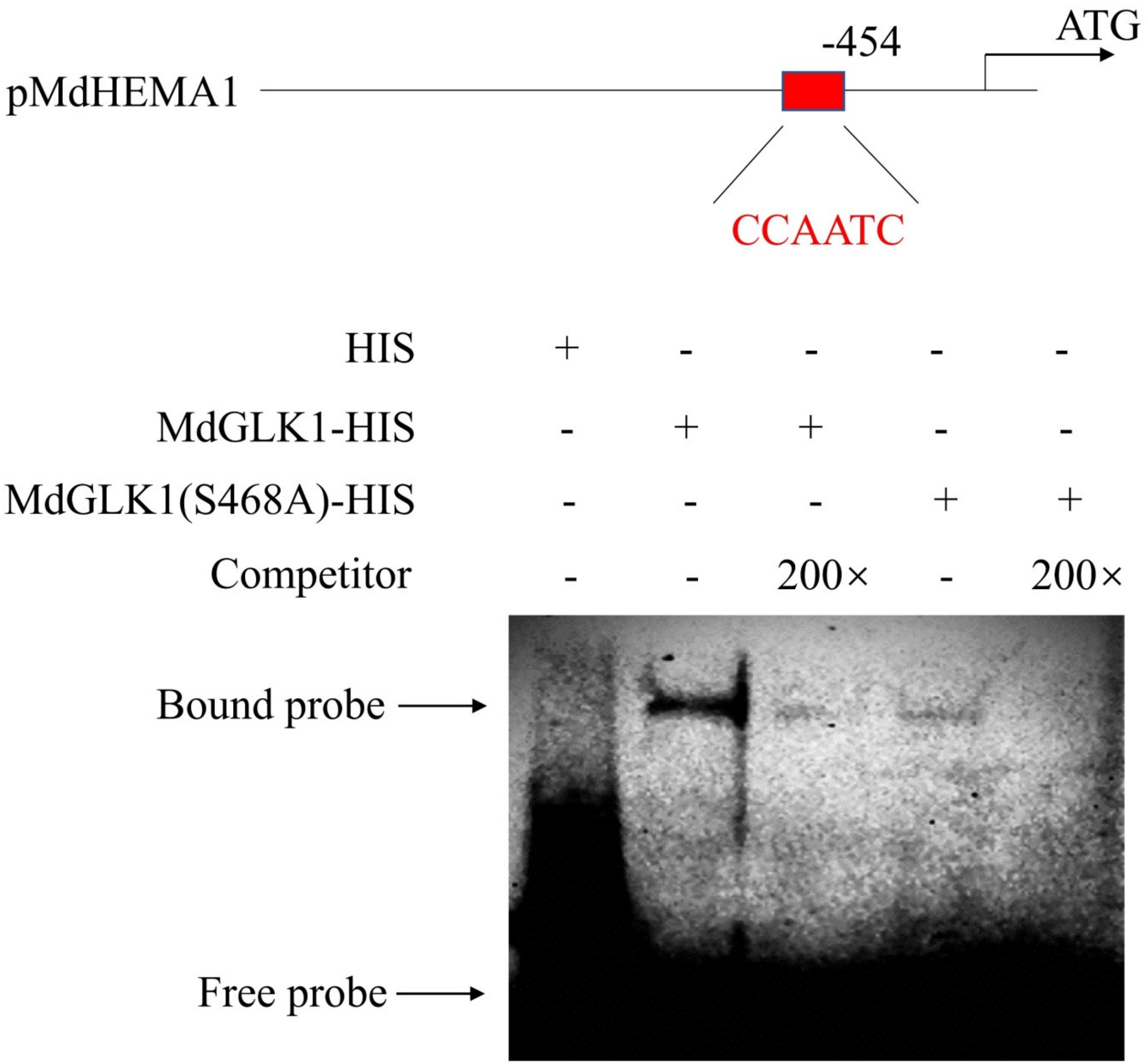

Supplemental Figure 9. EMSA detects MdGLK1-HIS and MdGLK1(S468A)-HIS binding to *MdHEMA1* promoter.

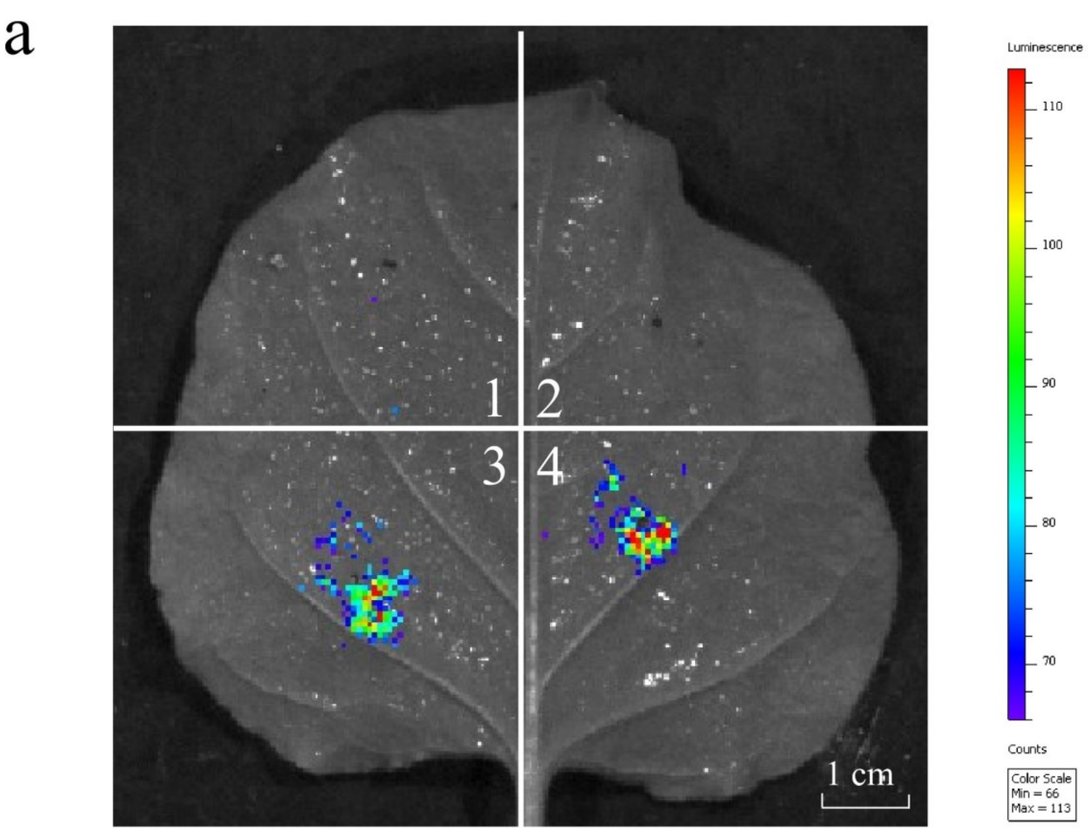

**LUC**<br>**35S$_{pro}$: 62-SK**<br>1	2<br>**LUC**<br>**35S$_{pro}$: MdGLK1(S468A)**
3<br>**MdHEMA1$_{pro}$:LUC**<br>**35S$_{pro}$: 62-SK**	4<br>**MdHEMA1$_{pro}$:LUC**<br>**35S$_{pro}$: MdGLK1(S468A)**

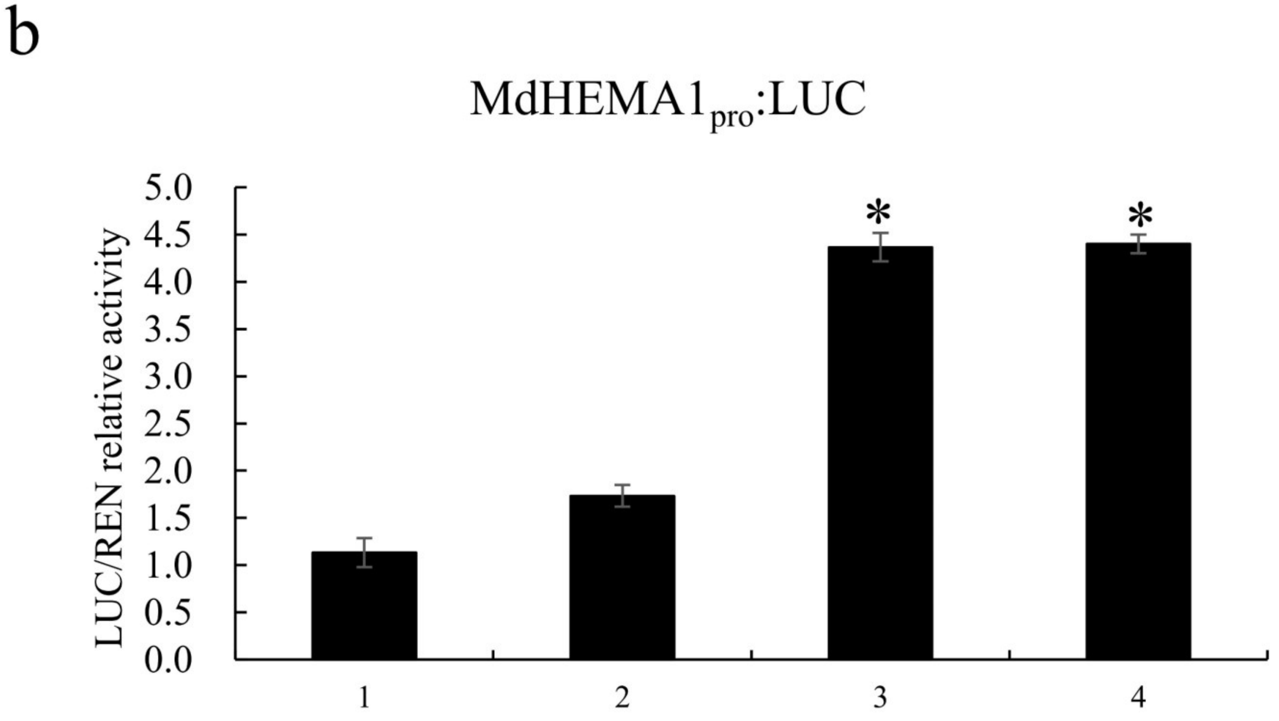

Supplemental Figure 10. The LUC validation of the effect of MdGLK1 phosphorylation site mutations on *MdHEMA1* expression. (a) The fluorescence signal of the effect of MdGLK1 phosphorylation site mutations on *MdHEMA1* expression. Scale bars, 1 cm. (b) The luciferase activity of the effect of MdGLK1 phosphorylation site mutations on *MdHEMA1* expression.

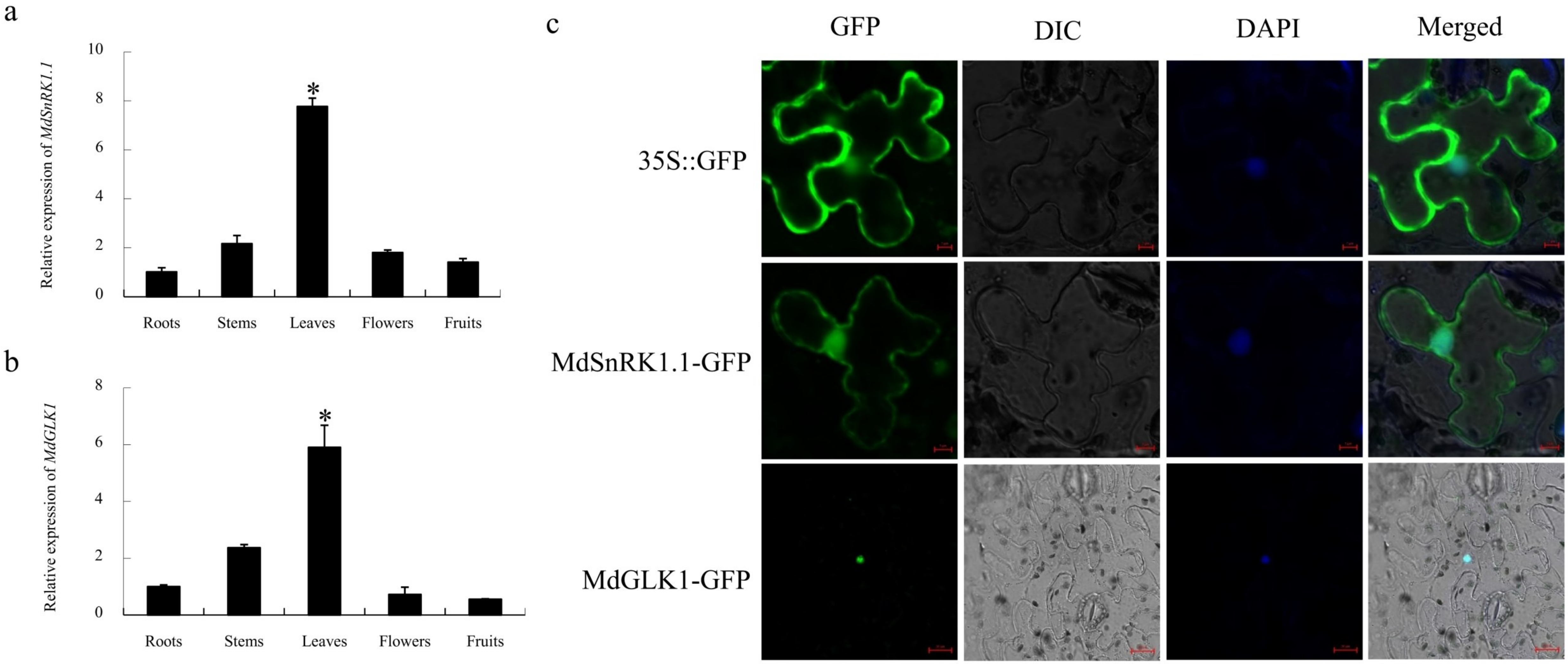

Supplemental Figure 11. Tissue expression patterns and subcellular localization of MdSnRK1.1 and MdGLK1. (a, b) The tissue expression patterns of *MdSnRK1.1* and *MdGLK1*. (c) The subcellular localization analysis of MdSnRK1.1 and MdGLK1. The expression level in roots might be set to 1. Values are mean ± SD of three biological replicate experiments and asterisks denote significant differences compared to the control: *$P < 0.05$; **$P < 0.01$. Scale bars, 5 μm.

Gene Name	Forward Primer	Reverse Primer
MdSnRK1.1(PRI)	GTCGACATGGATGGACCTGTTG	GGATCCCAAGGACGCGAAGTTG
MdGLK1(PRI)	GTCGACATGCTTCTTTTATCACCTTTG	GAATTCTCAAGTACAGGAGGGTGGAAA
MdSnRK1.1(AD)	GCGGATCCTGGATGGACCTGTTGG	GCGTCGACCTAAAGGACGCGAAGTTGG
MdGLK1(AD)	GAATTCATGCTTCTTTTATCACCTTTG	GTCGACAAGTACAGGAGGGTGGAAATT
MdGLK1-G1(AD)	GAATTCATGCTTCTTTTATCACCTTTG	GTGTTGATATCGTCGGCGGGTGCTGGAA
MdGLK1-G2(AD)	TGACGGAGACGTCTTGCCGGATTTGG	GTCGACAAGTACAGGAGGGTGGAAATT
MdGLK1-G3(AD)	ATCAACACAAAAACAACACCACCACC	GTCGACAAGTACAGGAGGGTGGAAATT
MdSnRK1.1(BD)	GAATTCATGGATGGACCTGTTG	GGATCCAAGGACGCGAAGTTG
MdSnRK1.1-S1(BD)	GGATCCTGGATGGACCTGTTGG	GTCGACGAACCATGCATGCTGACGAA
MdSnRK1.1-S2(BD)	GGATCAGGCTCATCTTCCTCGTTA	GTCGACCTAAAGGACGCGAAGTTGG
MdGLK1(BD)	GAATTCATGCTTCTTTTATCACCTTTG	GTCGACTCAAGYACAGGAGGGTGG
MdSnRK1.1(YFP)	GCGGATCCATGGATGGACCTGTTGG	GCGTCGACAAGGACGCGAAGTTGGG
MdGLK1(YFP)	GTCGACATGCTTCTTTTATCACCTTTG	GGTACCTCAAGTACAGGAGGGTGG
MdSnRK1.1 (nLUC)	GGTACCATGGATGGACCTGTTGG	GGATCC CTAAAGGACGCGAAGTTGG
MdGLK1(nLUC)	GTCGACATGCTTCTTTTATCACCTTTG	GAATTCAGTACAGGAGGGTGGAAAT
MdSnRK1.1 (cLUC)	GGATCCATGGATGGACCTGTTGGC	GAATTCCTAAAGGACGCGAAGTTGGG
MdGLK1(cLUC)	TCCCGGGGCCCCGGGGGTACCATGCTTCTTTTATCACCTTTG	GGGGAAATTCGAGCTCGAATTCTCAAGTACAGGAGGGTGGAAA
MdSnRK1.1(pET32a)	GGATCCATGGATGGACCTGTTG	GTCGACAAGGACGCGAAGTTG
MdSnRK1.1(pGEX)	GAATTCATGGATGGACCTGTTGGC	GTCGACCTAAAGGACGCGAAGTTGGG
MdGLK1(pCOLD)	GGTACCATGCTTCTTTTATCACCTTTG	GTCGACTCAAGTACAGGAGGGTGG
MdGLK1(S468A)	CTCGATTTTCATCCGGCAAAAGAAAGCA	TGCCTACTTTTAGCTCTTCTTCCACTC
pMdSnRK1.1(1300)	AAGCTTCTGTTTATCCAGCAGAACTTTG	TCTAGACGTACAAGAGATAACACCAG
pMdHEMA1(EMSA)	CGTTCCATTCCACCAATCAGAATGCAAAGC	GCTTTGCATTCTGATTGGTGGAATGGAACG
MdSnRK1.1(qRT)	TCCACCAGACACCATGCAACAAG	CACGAAGCGACTCAACCAGAAGG
MdSnRK1.2(qRT)	CAATTTCTTCCGAACTCAGTCC	CATCTTTCTGACCATCTCAGGA
MdSnRK1.3(qRT)	GAAGTGTAGGTGGGTTTTTGAC	CCAAAATAATGGTTACCGTGCA
MdABI1(qRT)	TGACGAGGATGAGTGCTTGAT	GACCTTCGCCGCCTTCTTA
MdABI2(qRT)	GACGACGAATGCCTAATT	TCTTGTGCCAGAGGAGTA
MdABI5(qRT)	GTATCAGAACGGCAACCA	GCATATCCATCACCACCA
MdGLK1(qRT)	CCGATACTGTCATGGTGGAGC	GAAGATCTAGAAAAATGACGGG
MdHEMA1(qRT)	GAACATGCACGCTCTTAAC	CGAGTTGAAGATTACACCAG
MdCAO(qRT)	CGACAAGCTAGGAGTGAGG	GTCCAAGCAAGCAGGTTG
MdCHLH(qRT)	TCCACCTTCTCACTCCTCAC	GAGAGGGAAGAAAGTTGGTC
MdACT(qRT)	GGATTTGCTGGTGATGATGCT	AGTTGCTCAGCCAAAGTTCTTGA
AtHEMA1(qRT)	CTGAGCGAGACCCTTGAGAATATGC	CTTCTGTTGTTGTTCCGCCATTGC
AtCAO(qRT)	GGTTCCGCCTTATAGTCCACACTTG	CACATCCTGGTTTCCCGTCTTCAC
AtCHLH(qRT)	CAGGGTCATACTCAGCCAACATCAG	GGAGCATCACTATCAAACGCAAACG
AtACT2(qRT)	AATTCCCCATGGGCA	TCATACTCGGCCTTGGA

Supplemental Table 1. Primer used for vector construction and gene expression analysis.
